# Integrated *In vitro* and *in vivo* evaluation of anticancer potentials of two clerodane diterpenes isolated from *Psidium guajava* (L.) through molecular docking approaches

**DOI:** 10.1371/journal.pone.0362655

**Published:** 2026-11-13

**Authors:** Most Chand Sultana Khatun, Mir Imam Ibne Wahed, Md. Jamal Hossain, Md. Ataur Rahman, Sabbir Ahmed, Md Hasibul Hasan

**Affiliations:** 1 Department of Pharmacy, Faculty of Life Science, Mawlana Bhashani Science and Technology University, Santosh, Tangail, Bangladesh; 2 Department of Pharmacy, Faculty of Science, University of Rajshahi, Rajshahi, Bangladesh; 3 Department of Pharmacy, School of Pharmaceutical Sciences, State University of Bangladesh, Dhaka, Bangladesh; Jadavpur University, INDIA

## Abstract

*Psidium guajava* (L.) is a medicinal plant rich in bioactive phytochemicals with promising therapeutic potential. This study aimed to isolate clerodane-type diterpenes from *P. guajava* and evaluate their anticancer activity through *in vitro* and *in vivo* assays supported by molecular docking.Two clerodane-type diterpenes, borapetoside F (**1**) and borapetoside B **(2**), were isolated from the ethyl acetate fraction of *P. guajava* and identified by ^1^H and ^13^C NMR spectroscopy. Cytotoxicity was evaluated against Ehrlich Ascites Carcinoma (EAC) cells using the MTT assay and in EAC-bearing Swiss albino mice, with bleomycin as the reference drug. Apoptosis was assessed by microscopy, DNA fragmentation, gene expression analysis. Compounds **1** and **2** demonstrated significant *in vitro* cytotoxicity, inhibiting EAC cell growth by 82.4% and 79.8%, respectively. In *in vivo* cytotoxicity assays, these compounds suppressed EAC cell proliferation by 61.26% and 48.88%, respectively. Optical and fluorescence microscopy revealed characteristic apoptotic morphological changes, while DNA fragmentation analysis confirmed apoptosis induction. Gene expression analysis demonstrated upregulation of the pro-apoptotic markers p53, Bax, and Cytochrome C, accompanied by downregulation of the anti-apoptotic genes Bcl-2 and NF-κB. Molecular docking further supported the experimental findings by demonstrating favorable interactions of both compounds with apoptosis-related target proteins. Collectively,. Borapetoside F (**1**) and borapetoside B (**2**) exhibit significant anticancer activity by inhibiting EAC cell proliferation and inducing apoptosis, suggesting their potential as promising lead compounds for anticancer drug development.

## 1. Introduction

*Psidium guajava* L. (*P. guajava*) is an evergreen shrub-like tree belonging to the Myrtaceae family which cultivated in different countries mainly in tropical and subtropical areas [1]. In Bangladesh and other South Asian countries, guava leaves and fruits are frequently used in folk medicine for gastrointestinal, metabolic, and inflammatory disorders. Phytochemical investigations of *P. guajava* have revealed a broad spectrum of bioactive secondary metabolites, including phenols, flavonoids, triterpenoids, alkaloids, tannin, essential oil, sesquiterpene alcohol and vitamins that are largely responsible for its reported antioxidant, antimicrobial, anti-diabetic, anti-inflammatory, hepatoprotective, and anticancer activities [2, 3]. *P. guajava* fruits are preferred to make very popular healthy food products such as juice, jam, jelly, liquors and toffee [4]. *P. guajava* used for their traditional medicinal values in the treatment of cough, insomnia, diarrhea, hypertension, obesity and diabetes [5–7]. Guava leaves contain a wide range of bioactive chemical constituents, including guaijaverin, quercetin, avicularin, myricetin, hyperin, kaempferol, apigenin, gallic acid, rutin, chlorogenic acid, pyrogallol, isoquercitrin, caffeic acid, luteolin, morin, prodelphinidin dimer isomer, pedunculagin, casuariin, vanillic acid, ellagic acid, myrciaphenone B, vescalagin [8–13]. Besesides, guava leaf extract is also used as anti-cestodal [14], antimicrobial [15], analgesic, anti-inflammatory and antioxidant agents [16,17]. Guava leave extract contains flavonoids such as quercetin which have spasmolytic, antiviral and antitumor activities [18–20], therefore, the leave part of the plant has been selected for the present study. The discovery of novel anticancer compounds from natural sources has received considerable attention in recent decades [21]. Researchers dem6onstrated that guava seed polysaccharides have the potential for anticancer activity of against Michigan Cancer Foundation-7 (MCF-7) cells. This result revealed a significant dose-dependent inhibition of MCF-7 cell viability [22]. Previous studies have demonstrated that antioxidant, antimicrobial, antidiabetic, and anti-inflammatory activities of different fractions of *P. guajava* leaf extracts have been well documented [23]. Although there have been many pharmacological studies on *P. guajav*a, few have looked at isolating bioactive secondary metabolites from guava leaves and further evaluating their anticancer properties. There are few studies that combine *in vitro, in vivo*, and molecular docking methods to investigate EAC-induced breast cancer. In this context, the present study was undertaken to isolate and characterize secondary metabolite from the ethyl acetate fraction of *P. guajava* leaves and evaluation of cytotoxicity potential through *in vitro* MTT assay and *in vivo* assessment against Ehrlich Ascites Carcinoma (EAC) cells inducing breast cancer.

## 2. Materials and methods

### 2.1. Plant materials

*P. guajava* leaves were collected in July 2022 and the National Herbarium confirmed the authenticity of the plant. They gave a voucher specimen no. DACB-55126 for further reference.

### 2.2. Materials and instruments

Bleomycin, DME medium (Dulbecco’s Modified Eagle Medium), 10% fetal bovine serum, MTT (3- [4, 5-dimethylthiazol-2-yl]-2,5- diphenyl tetrazolium bromide) and EAC cells were supplied by authorized suppliers. Trinocular microscope with camera (Olympus, Japan), biological biosafety cabinet (NU-400E, Nuaire, USA), hemocytometer (Nexcelom, USA) and CO_2_ incubator (Nuaire, USA) were used to execute the *in vitro* and *in vivo* anticancer test., Dimethyl sulfoxide (DMSO), 4’ 6-diamidino-2-phenylindole (DAPI), trypan blue dye, sodium carbonate, ALT (Alanine Aminotransferase) AST (Aspartate aminotransferase) and ALP (Alkaline Phosphatase) were purchased from Merk (Darmstadt, Germany). A Burker AMX-400 operating at 400 MHz and 100 MHz were used to measure for ^1^H-NMR and ^13^C spectra respectively. Tetramethyl silane (TMS) as internal reference was used to measure chemical shift expressed as in δ (ppm) scale and coupling constants (*J*) are expressed in Hertz (Hz). Analytical thin layer chromatography (TLC) was performed by using pre-coated TLC (Silica gel 60 F_254_-Merk KGa, Dermstadt, Germany) and plates were observed under UV light (254 nm). For the separation of compound, silica gel packed column chromatography technique was used (Kieselgel 60, 230–400 mesh, Merck KGaA, Dermstadt, Germany). Preparative TLC plates were prepared by glass (20 × 20 cm) surface coated with slurry of silica gel.

### 2.3. Extraction and fractionation

The dried powdered leaves (1 kg) were soaked in methanol (3L) and were kept at room temperature for 7 days with occasional shaking and stirring. The extraction was filtered through cotton plug and then using Whatman filter paper no. 1. The liquor was evaporated by rotary evaporator to get highly concentrated crude extract. The semisolid mass (50g) was fractionated by *n*-hexane, chloroform and ethyl acetate according to the Kupchan modified partitioning protocol [24] and subsequently the fractions were dried to give 3.1 gm *n*-hexane (HPG), 6.3 gm chloroform (CPG) and 7.5 gm ethyl acetate (EPG) semisolid mass (% yield of HPG, CPG EPG are 6.2%, 12.6% and 15% respectively) respectively which were kept in a refrigerator until further use.

### 2.4. Isolation of chemical constituents

The phytochemical analysis of EPG fraction confirmed the presence of flavonoids, phenols and alkaloids. Therefore, EPG fraction was column chromatographed for the separation and isolation of pure compounds. The column sample was prepared by dissolving the EPG fraction (2g) in methanol which was thoroughly mixed with small amount of silica gel (mesh size 60–120) and then air dried to obtain coarse sample. The dried sample poured into the column (Length 600 mm & diameter 40 mm), reasonably packed and eluted with a solvent system comprised of *n*-hexane and ethyl acetate (9:1) which was applied through the column in order of increasing polarities. The eluted solvent sample was collected in a series of glass tubes (10 ml each) which were checked for similarities by TLC; and the fractions of similar characteristics were combined to yield twelve fractions (1–12) [25]. Among the combined fractions, 7 and 8 were chromatographed by preparative thin layer chromatography (PTLC) by using the solvent systems of *n*-hexane: ethyl acetate (8.5:1.5) and *n*-hexane: ethyl acetate (8:2) respectively. Consequently, compound **1**, yellow amorphous powder (25.7 mg) known as borapetoside F and compound **2**, white powder (30.3 mg) known as borapetoside B were obtained from fractions 7 and 8 respectively.

### 2.5. Properties of isolated compounds

**Borapetoside F:** Yellow amorphous powder, ^1^H NMR (400 MHz, CD_3_OD), δ7.7 (1H, *d, J* = 1.4 Hz, H-16), 7.5 (1H, *d, J* = 2.3 Hz, H-15), 6.8 (1H, *d, J* = 8.1 Hz, H-7), 6.78 (1H, *m,* H-3), 6.5 (1H, *s,* H-14), 5.34 (1H, *dd, J* = 11.2 and 15.4 Hz, H-12), 4.55 *(s*, 1H, H-6), 4.5 (1H, *d, J* = 7.6 Hz, H-1’), 3.2 (1H, *m,* H-2’), 3.38 (1H, *s*, H-5’), 3.88 (2H, *m,* H-6’), 2.4 (2H, *m,* H-2), 2.3 (2H, *m,* H-11), 2.2 (1H, *m,* H-10), 1.3 (3H, *s,* H-19), 1.2 (3H, *s,* H-20), 3.67 (3H, *s,* OCH_3_−18). ^13^C NMR (100 MHz, CD_3_OD): δ 170.2 (C-18), 142.0 (C-15), 141 (C-16), 137.5 (C-7), 136.3 (C-4), 123.3 (C-13), 108.4 (C- 14), 105.2 (C-1’), 78.4 (C-3’), 70(C-4’), 79 (C-5’), 62 (C-6’), 50.2 (OCH3−18), 47.3 (C-11), 38.4 (C-5), 37.3 (C-9), 26.1 (C-19), 25.2(C- 2), 25.2 (C-20), 19.5(C- 1).

**Borapetoside B:** White amorphous powder, `H NMR (400 MHz, CD_3_OD), δ7.4 (1H, *d, J* = 1.4 Hz, H-16), 7.2 (1H, *d, J* = 2.3 Hz, H-15), 6.4 (1H, *s,* H-14), 6.3 (1H, *d, J* = 4.4 Hz H-3), 5.4 (1H, *dd, J* = 11.2 and 15.4 Hz, H-12), 4.55 *(s*, 1H, H-6), 4.3 *(*1H, *s*, H-2), 4.41 (1H, *d, J* = 7.6 Hz, H-1’), 3.6 (2H, *m,* H-6’), 3.25 (1H, *s*, H-5’), 3.18 (1H, *m,* H-2’), 1.9 (2H, *m,* H-11), 2.2 (1H, *m,* H-10), 1.4 (1H, *s,* H-19), 1.2(3H, *s,* H-20), 3.65 (H, *s,* OCH_3_−18), 2.11 *(*2H, *m*, H-1),. ^13^C NMR (100 MHz, CD_3_OD): δ 178 (C-17), 168.3 (C-18), 145.4 (C-15), 140.1 (C-16), 142.4 (C-1), 139.6 (C-4),- 124.4 (C-13), 107.2 (C- 14), 105.5 (C-1’), 77.2 (C-3’), 70 (C-4’), 77.1 (C-5’), 62.3 (C-6’), 79.2 (C-6), 70.3 (C-12), 66.4 (C-2), 50.2 (OCH3−18), 48.6 (C-10), 47.3 (C-11), 44.3 (C-5), 42.1 (C-8), 38.1 (C-9), 26.1 (C-19), 25.2 (C-20), 27.3(C- 2).

### 2.6. Experimental animals

Female *Swiss albino* mice (average 25g and number, 30) were used for these experiments which were collected from the authentic source, Rajshahi University, Bangladesh. The animals used in the *in vivo* anticancer experiment were conducted in accordance with institutional ethical guidelines and approved protocols. Humane endpoints were predefined to minimize animal suffering. Animals would be humanely euthanized if they exhibited any of the following criteria: severe weight loss (>20% of initial body weight), persistent lethargy, inability to access food or water, abnormal posture, labored breathing, or signs of severe pain or distress unresponsive to intervention. But we were not observed any of the criteria. All animals were lived at the end of the experiment and euthanized after finishing the experiment.The total duration of the *in vivo* anticancer test was 15 days. The first 7 days were allocated for acclimatization of the animals to the laboratory environment, allowing stabilization of physiological conditions and attainment of appropriate body weight to meet the experimental criteria. After 24 hour of tumor inoculation, treatment was carried-out for 5 days and on the six day mice were sacrificed. Animal health and behavior were monitored daily, including assessment of body weight, food and water intake, physical appearance, and behavioral changes. All efforts were made to minimize suffering and distress. Animals were housed under standard laboratory conditions with controlled temperature, humidity, and a 12-hour light/dark cycle, with free access to food and water. Environmental enrichment was also provided to promote well-being. All animals were humanely euthanized at the designated experimental endpoint in accordance with approved ethical guidelines. No animals died during the course of the study. Efforts were made to minimize animal suffering and distress at all stages of the experiment. Before sacrificing, the mice were anesthetized by phenobarbital. The powder form of phenobarbital was dissolved with normal saline (0.9% NaCl) to make the concentration 5%. The dose was 125 mg/kg body weight of mice and the anesthetic duration time was about 35 minutes out from the peritoneal cavity by 0.98% normal saline.

### 2.7. *In vitro* cytotoxicity test

Compounds **1** and **2** were used to execute *in vitro* cytotoxicity test against EAC cell by MTT colorimetric assay [26]. The RPMI-1640 media was taken in each well of a 96-well culture plate to culture 200 μl of EAC cells (1 × 106 cells) at various concentrations (500, 250, 125, 62.5, and 31.25 μg/ml) of the two compounds. Then the culture plate was incubated at 37°C with constant flow of 5% CO_2_ and then removed the supernatant. After that 180 μl of PBS and 20 μl of MTT were added to each well of the culture plate followed by incubation at 37°C for 8 hrs. Again, removed the supernatant and added 200 μl of acidic isopropanol into each well which was again incubated at 37°C for 1 hr. Finally, a multiwell plate was used to examine the plate at 570 nm. This test was done for each sample three times.

### 2.8. *In-viv*o anticancer test against Ehrlich Ascites Carcinoma (EAC) cells

#### 2.8.1. Transplantation of tumor.

EAC cells were collected and maintained according to the standard protocol [27]. Briefly, the EAC cells were cultured in mice by intra-peritoneal transplantation and aspirated from the peritoneal cavity. The freshly cultured EAC cells were washed, diluted (0.9% normal saline), counted and analyzed for viability using a hemocytometer (1x10^6^cells/ml).

#### 2.8.2. *In vivo* cell growth inhibition.

The cells growth inhibition of Compound **1** and compound **2** were observed in mice following standard method [28]. On day 0, before treatment 1x10^6^ cells were inoculated in mice and after 24 hours of inoculation mice were divided into 5 groups where mice received neither EAC cells nor any treatment considered as normal control or Group I, Group II received vehicle used as disease control, Group III received pure bleomycin (0.3 mg/kg/day) considered as standard, Group IV and Group V received compound **1** and Compound **2** (5 mg/kg/day) for 5 days. All mice of Group II-V were bearing EAC cells. On day 6, the volume of tumor was about 98 mm^3^ and in this experimental model, tumor volume is usually estimated, not directly measured, using caliper measurements of tumor dimensions.Besides,on day 6, mice from each group were sacrificed, and tumor cells were collected from peritoneal cavities which were then harvested using normal saline (0.9%). Finally, the viable EAC cells from different groups of mice were stained with Typhan blue and counted using a hemocytometer. The following formula was used to calculate growth inhibition (%):


$$\begin{array}{c} \%\text{\textasciitilde{}cell\textasciitilde{}groeth\textasciitilde{}inhibition\textasciitilde{}}=\text{\textasciitilde{}(1-T/C)\textasciitilde{}}\times \text{\textasciitilde{}}100 \end{array}$$

Where, T = treatment group; C = control group

### 2.9. Assessment of apoptosis by DAPI staining

The EAC cells collected from both control and treated groups were stained with DAPI (0.1 μg/ml) in dark condition at 37°C for 15 min [29]. The cells were washed with PBS (Phosphate buffer saline), re-suspended and viewed under microscope. For morphological and cellular study, supernatant (5μl) was taken into slide and examined with the help of optical and fluorescence microscope (Olympus IX71, Korea).

### 2.10. DNA fragmentation assay

DNA fragmentation assay was done according to the method described earlier [30]. EAC cells from both control and treated groups were collected after consecutive days of treatment. DNA isolation kit (TIANGEN Biotech, Beijing, China) was used to isolate genomic DNA and 1% agarose gel was utilized to analyze DNA under gel documentation system (Alphaimager Mini System, ProteinSimple).

### 2.11. RNA isolation and cDNA synthesis

RNA simple Total RNA Kit (Tiangen, Beijing, China) was used to isolate RNA. The concentration and purity of RNA was determined by spectrophotometer (Nanodrop 2000 spectrophotometer, Thermo scientific). Isolated RNA about 3 μg were converted to cDNA which is a reaction mixer containing 5 × 1st strand buffer (4 μL), M-MLV reverse transcriptase (1 μL), dNTPs (2 μL), oligo dT (2 μL) and desired amount of dH2O to make 20 μL volume [31].

### 2.12. PCR amplification of genes related to apoptosis

Gene expression analysis was performed by Real-time polymerase chain reaction (real-time PCR) technique [32]. The gene specific oligos were 25-μL reaction mixtures consisted of 1X Taq polymerase buffer, 25 pmol each of forwarded and reverse primers, 2.5 mM of each dNTPs, and 0.25 μL of platinum Taq polymerase (Tiangen, Beijing, China). GAPDH, P^53^, Bax, (proapoptotic gene) BcL-2 and NF- κB (antiapoptotic gene) consequent oligonucleotide (IDT, Singapore) were used; and their thermal condition and prime sequence were included in Table 1. To compare the gene expression patterns, GAPDH, a housekeeping gene was used as reference. 1% agarose gel with a fluorescent dye Ethidium Bromide (EtBr) was used for separation of the PCR product of these Oligos and GAPDH. Lastly, UV-trans illuminator (Protein Simple) was used to observe the image.

**Table 1:** Prime sequence and PCR protocol used for the RT-PCR assay.

Target genes	Prime sequence	AT, amplification (bp)	PCR protocol
GAPDH (F)	5′-GTGGAAGGACTCATGACCACAG-3′	52 ^0^C, 350 bp	Program was set at 95 ^0^C for 3 mins, 95 ^0^C for 1 min, 5258^0^C (changeable annealing temperature for each oligo) for 1 min, 35 cycles followed by 72 ^0^C for 1 min, 72 ^0^C for 10 min, and finally hold at 72 ^0^C
GAPDH (R)	5′-CTGGTGCTCAGTGTAGCCCAG-3′		
P^53^ (F)	5′-GCGTCTTAGAGACAGTTGCCT-3′	53^0^C, 500 bp	
P^53^ (R)	5′-GGATAGGTCGGCGGTTCATGC-3′		
BcL-2 (F)	5′-GTGGAGGAGCTCTTCAGGGA-3′	53 ^0^C, 150 bp	
BcL-2 (R)	5′-AGGCACCCAGGGTGATGCAA-3′		
Bax (F)	5′-CGCCCACCAGCTCTGAGCAGA-3′	50 ^0^C, 350 bp	
Bax (R)	5′-GCCACGTGGGCGTCCCAAAGT-3′		
Cyt-C (F)	5′-CCAGGTATACAAGCAGGTGTGCTC-3′	50 ^0^C, 150 bp	
Cyt-C (R)	5′-CATCATTAGGGCCATCCTGGAC-3′		
NF-κB (F)	5′-AACAAAATGCCCCACGGTTA-3′	55 ^0^C, 125 bp	
NF-κB (R)	5′-GGGACGATGCAATGGACTGT-3′		

**AT=annealing temperature; F = forward; PCR = polymerase chain reaction; R = reverse; RT-PCR = reverse transcriptase polymerase chain reaction**.

### 2.13. Hematological, liver and kidney function test

Mice from each group were sacrificed and blood samples were collected for the estimation of markers of liver and kidney functions. The blood parameters including RBC, total WBC and percentage of hemoglobin (% Hb) were determined by standard protocol [33].

### 2.14. Molecular docking study

The widely used software packages (PyRx, PyMoL 2.3, Discovery Studio 4.5, and Swiss PDB viewer) were used to perform docking of the two isolated compounds.

#### 2.14.1. Gene preparation.

To assess anticancer activities apoptotic gene Bax (PDB ID 5W60), Cyt-C protein (PDB ID: 2B4Z) and anti-apoptotic gene Bcl-2 (PDB ID: 2W3L), NF-kB (PDB ID: 1NFK) were selected from protein data bank [34] due to their key role in apoptosis. PyMOL (version 1.3) software was used to remove hetero atom and water molecules from each gene structure which may hinder drug protein interaction [35].

#### 2.14.2. Ligand preparation.

The structure of the compounds **1** and **2** were found in the PubChem database and the standard drug bleomycin was used to compare the binding affinity of the target molecules were downloaded and saved in 3D SDF format. Discovery studio 4.5 was used to load the ligand serially with their PubChem CID. To enhance the docking accuracy, all the phytochemicals were optimized via Pm6 semi-empirical method [36,37].

#### 2.14.3. Docking.

Docking study was conducted by using AutoDockVina software [38].To perform docking the center grid boxes were located on the midpoint of the protein against ligands bleomycin and compounds **1** and **2.** According to binding pocket prediction and literature hunt [39] the grid box size in x, y, and z directions was determined. The docking pose was captured by using PyMOL and Accelrys Discovery Studio 4.5 and the lowest binding free energy was considered among the selected proteins [40]. Representative three-dimensional (3D) binding poses and two-dimensional (2D) interaction diagrams were generated for compounds 1, 2, and bleomycin against the selected apoptosis-related target proteins. For Bax and Bcl-2, where co-crystallized ligands were available, the ligands were removed from the respective protein structures and redocked into their original binding sites using the same grid parameters to validate the docking protocol. The predicted and experimentally determined ligand orientations were compared based on root-mean-square deviation (RMSD) (< 2.0 Å) After validation, the docking protocol was applied to evaluate the binding modes of compounds **1, 2**, and bleomycin. The predicted poses were then compared with the co-crystallized ligands of selected proteins to assess binding-site occupation and interaction patterns.

### 2.15. Ethical permission

This study was conducted in full compliance with the guidelines outlined in the National Institutes of Health’s Guide for the Care and Use of Laboratory Animals. Animal study was approved by the Ethical Review committee (ERC), Mawlana Bhashani Science and Technology University (Reference number-MBSTU/ERC/ECC/157/45/2026).

### 2.16. Statistical analysis

Values are expressed as mean ± SEM. Differences between two groups were measured by one-way analysis of variance (ANOVA) followed by Dunnet test where p < 0.05 were considered statistically significant. The *in vitro* and *in-silico* experiments were conducted in MS Excel 2019 version where below 1% were considered for the standard errors.

## 3. Results

### 3.1. Identification and characterization of isolated compounds

EPG fraction was used to isolate pure compounds **1** and **2** by column chromatography techniques (Fig 1.).

**Fig 1:**
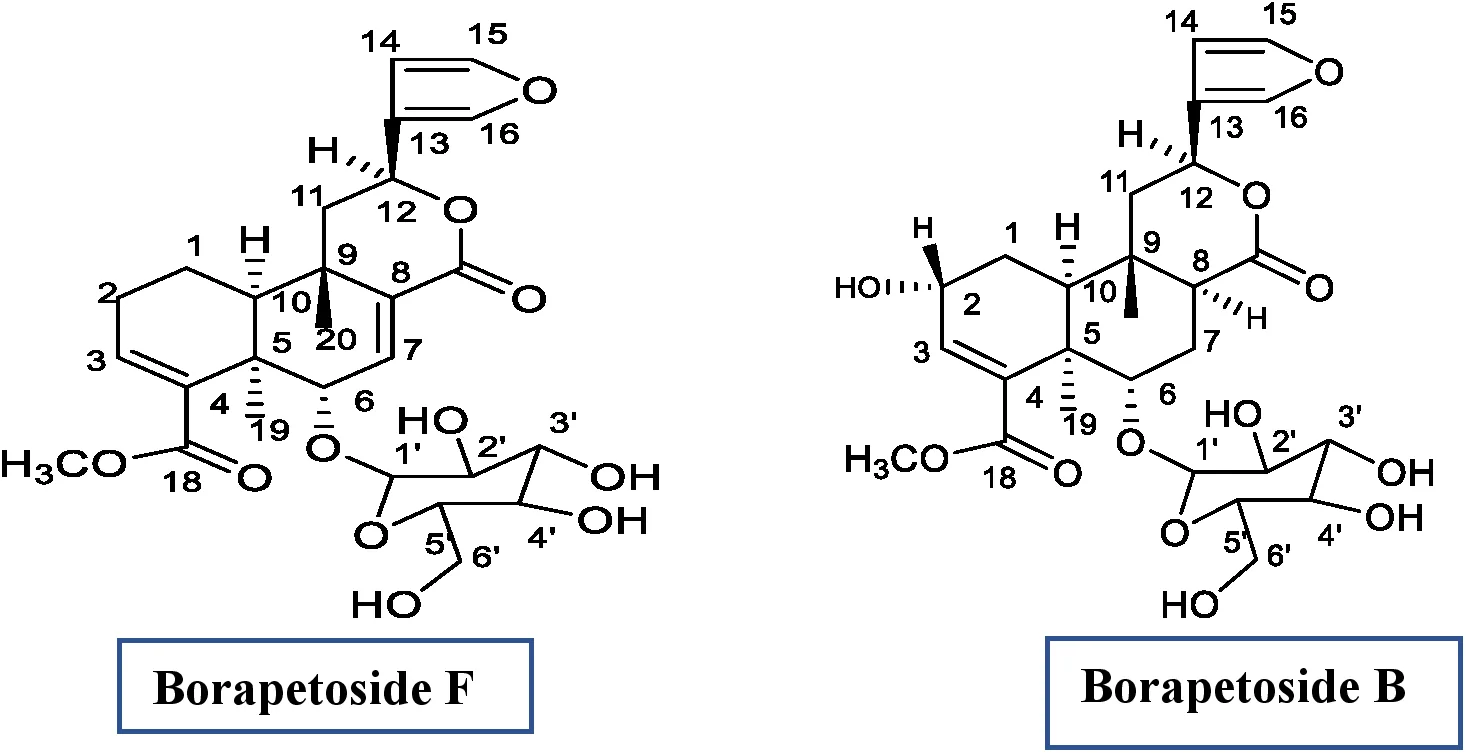
Chemical structure of compounds 1 and 2 isolated from *P. guajava.*

Compound **1** was obtained from ethyl acetate fraction of *P. guajava* as a yellow amorphous powder which turns reddish black followed by spraying with vanillin- sulfuric acid and heating. The ^1^H NMR of compound **1** has been shown that a β-mono-substituted furan ring indicated by the ring at δ 6.5, 7.5, 7.7 ppm and at δc108.4, 142, and 141 (**S1 Fig**). Two singlets were observed for two angular methyl groups integrating at δ 1.3 and 1.2 corresponding to H-19 and H-20 and at C-3 and C-4 position a tri-substituted double bond was found. The coupling constant, J = 7.6 Hz resonate at 4.5 in the ^1^H NMR spectra due to anomeric protons of the glucopyranose which suggested that the compounds contain glycosidic linkages with β-configuration. Moreover, a methoxycarbonyl group (δ 3.67 and δc 50.2) present at the position of C-4 and a lactone ring between C-8 (δc 50.2) and C-12 (δc 50.2) (S1 Fig). The **1**H NMR of compound **2** is also comprised β- mono-substituted furan ring, two angular methyl groups, glycosidic linkages for glucopyranose, a lactone ring which gave a very similar peak of compound **1**. The only difference is that at the position of 2, compound **2** contains a -OH group resonate at δ 4.3 ppm, and it does not contain double bond between 7 and 8 position (δc 42.1, C-8) (S2 Fig). where in compound **1**, it gave signal at δc 137.5 (C-7) because an aromatic double bond is present between 7 and 8. The biogenic properties such as two angular methyl linked to C-5 and C-9, two ring junction methines at C-8 and C-10 and a methoxycarbonyl group is attached to C-4 [41] indicate that both compound **1** and **2** are clerodane type bicyclic diterpenes. Based on all spectral evidence indicate that compound **1** and compound **2** identified as borapetoside F and borapetoside B (S2 Fig) [42]. Borapetoside F and boraptoside B are furanoid diterpene glycosides of the clerodane type. Previously, these two diterpene glycoside have been isolated from *Tinospora rumphii* and demonstrated to have various pharmacological properties [43].

### 3.2. *In vitro* anticancer activity

*In vitro* cytotoxicity of compounds **1** and **2** against EAC cells have shown that (Fig 2) both compounds significantly decreased EAC cell growth as compared to standard bleomycin. At the dose of 500 μg/ml, compounds **1** and **2** showed inhibition of EAC cell growth of 82.4% and 79.8% respectively, whereas the standard drug bleomycin inhibited cell growth of 90.7% at the dose of 250 μg/ml. Moreover, the IC_50_ values of the compounds **1** and **2** were 83 μg/ml and 136.11 μg/ml compared bleomycin which was 31.3 μg/ml (S1 Table and S3 Fig).

**Fig 2:**
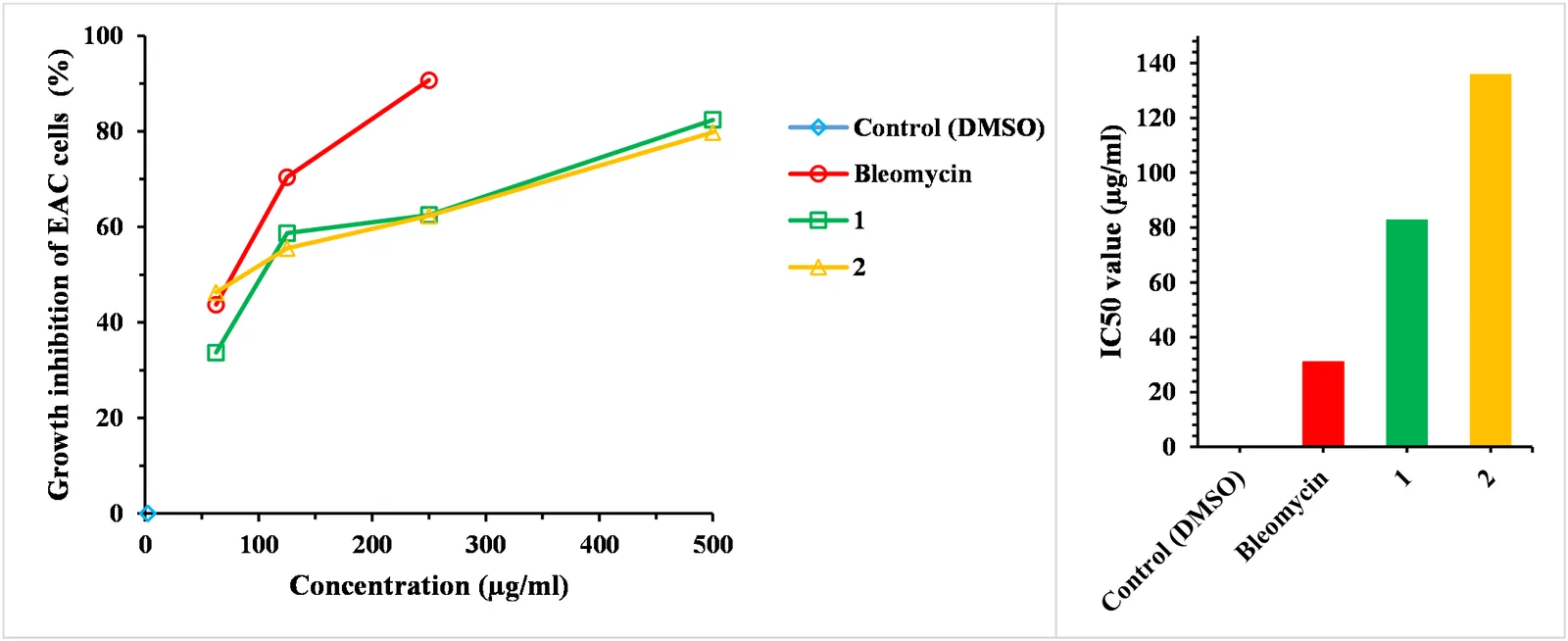
*In vitro* cytotoxic activity of compounds 1 and 2 on EAC cell line.

### 3.3. In vivo cell growth inhibition

*In vivo* anticancer activity of compounds **1** and **2** against EAC cells (Table 2) revealed that compounds **1** and **2** demonstrated significant reduction in viable EAC cells count compared to control group. Moreover, the cell growth inhibition of compounds **1** and **2** were 61.26% and 48.88% respectively. The inhibitory effect of compounds **1** and **2** on tumor cell proliferation was comparable to that of the standard anticancer agent bleomycin.

**Table 2:** *In vivo* cell growth inhibition of compounds 1 and 2 against EAC cell.

Groups	Drug	Dose	Viable EAC cells (x10^6^cells/ml)	Cell growth inhibition (%)
Group I		--	33 ± 14.3	--
Group II	DMSO		315 ± 22.2	--
Group III	Bleomycin,	0.3mg/kg	45 ± 17.3*	85.71
Group IV	Compound **1**	5mg/kg	122 ± 19.4*	61.26 (61.3)
Group V	Compound **2**	5mg/kg	161 ± 23.2*	48.88 (48.9)

Data are expressed in mean ±SEM at significant value of p < 0.05 and compared with control group.

### 3.4. Detection of apoptotic EAC cells by DAPI staining

The morphological changes of EAC cells were observed using DAPI after 5 days of treatment. The nuclei of EAC cell from control group were regular, round and gently stained as seen in fluorescence microscope. On the other hand, chromatin condensation, fragmented DNA and membrane abscess were found in compounds **1** and **2,** which is the hallmark of apoptosis [44]. In optical microscope, the number of cells decreased in treatment groups (Group III, Group IV and Group V) compared to the control group (Group II) in where the number of cells were more and uniform in shape. Besides, cells in Group I displayed normal size, number and morphology, suggesting a healthy cellular appearance. The results indicated that compounds **1** and **2** posses the potential to induce DNA damage leading to apoptosis (Fig 3).

**Fig 3:**
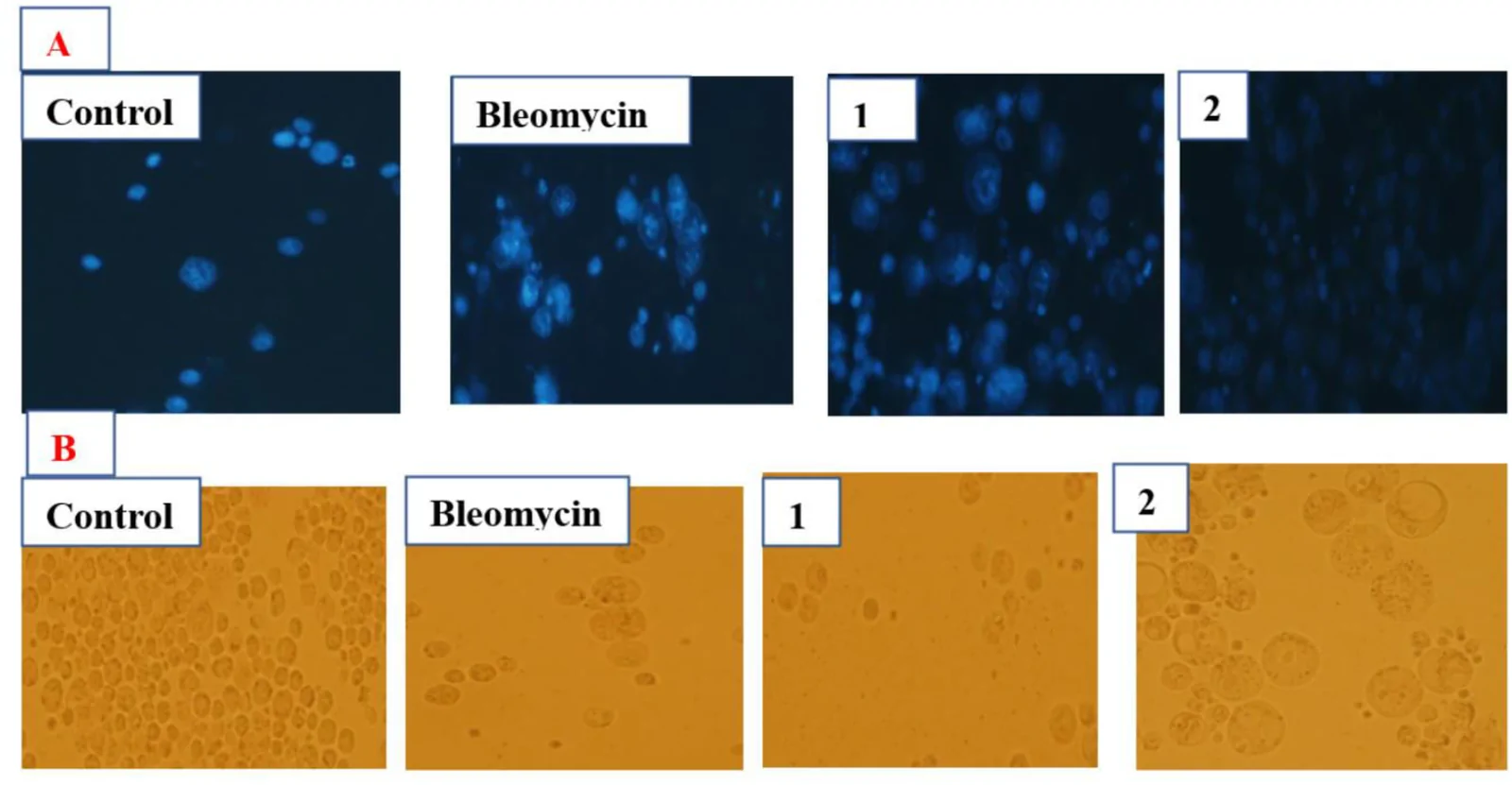
Displaying DAPI staining image of all groups of mice in Fluorescence microscope (A) and Optical microscope (B) on EAC cells. Both Fluorescence microscope (A) and Optical microscope (B) represented compounds **1** and **2** induced apoptosis.

### 3.5. DNA fragmentation assay

The EAC cells from control and treatment groups (Group II, III, IV and V) were used to perform DNA fragmentation assay (Fig 4). The EAC cells treated with compounds **1** and **2** were found smeared, fragmented and ladder type of DNA, whereby chromatin condensation was also observed. On the contrary, control groups represent unfragmented, uniform and distinct bands (Fig 4).

**Fig 4:**
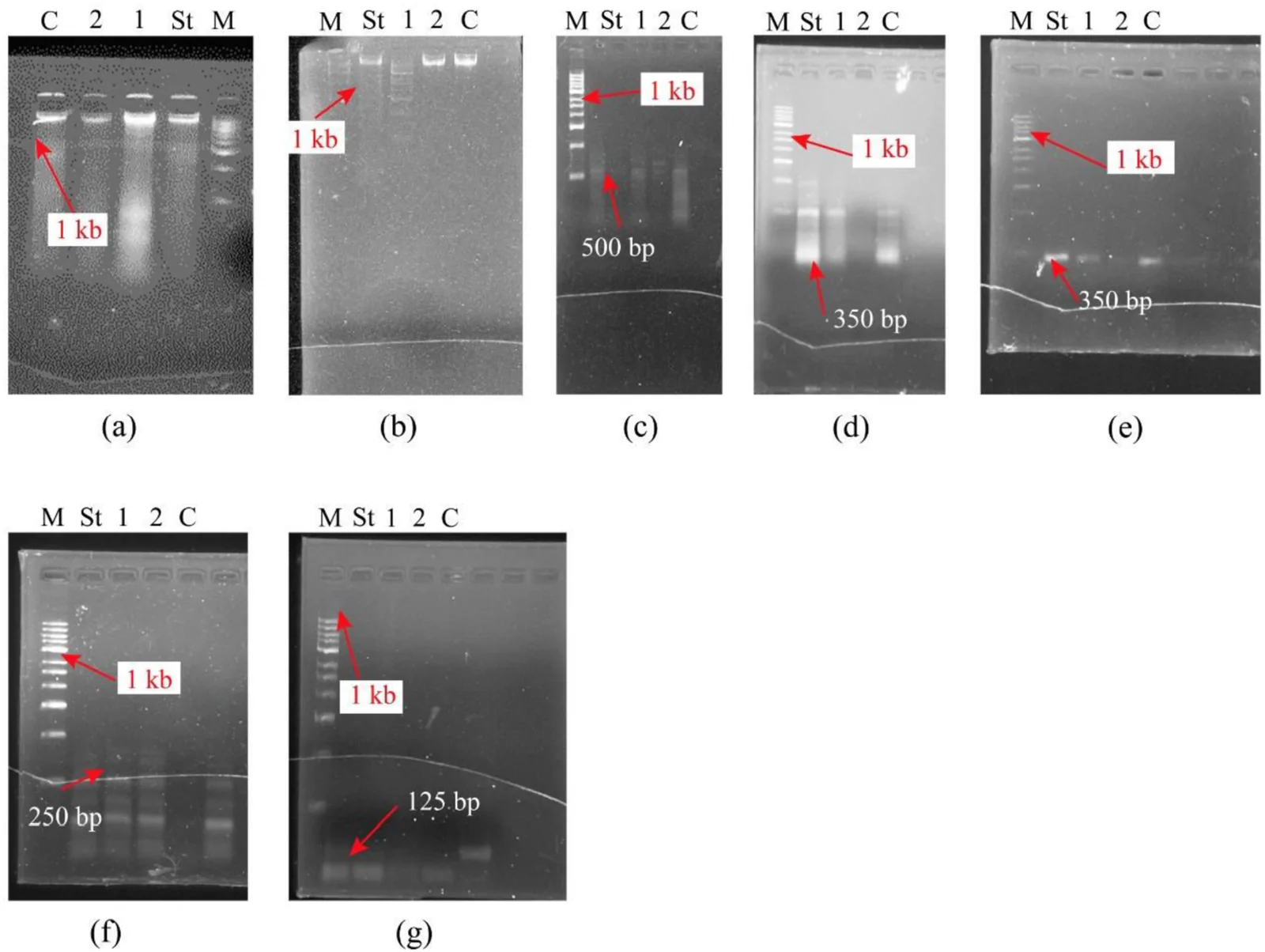
Pro-apoptotic and anti-apoptotic genes expression by semi-quantitative RT-PCR. Lane (M), (C), (**1** & **2**) represent molecular marker, control and compounds **1** and **2** respectively. Here (a) Genomic DNA fragmentation (b) housekeeping gene GAPDH expression as a references; (c, d, and e) Pro-apoptotic gene P53, Bax and Cyt-C upregulation; (f, g) anti-apoptotic gene Bcl-2 and NF-κB down regulation in different groups.

### 3.6. Modulation of apoptotic gene expression

The expression of apoptotic genes p53, Bax (proapoptotic), Cyt-C and anti-apoptotic Bcl-2, NF- κB was determined by RT-PCR analysis (Fig 4). Here GAPDH (housekeeping gene) was used as reference gene. The overexpression of tumor suppressor gene p53 was observed in compounds **1** and **2** treated cells at the 500 bp position on the gel. Similarly, treatment with compounds **1** and **2** resulted in upregulated expression of the proapoptotic genes Bax and Cyt-C with corresponding bands at approximately 350 bp. In contrast, a marked downregulation of the anti-apoptotic genes Bcl-2 and NF-κB in treated cells and their corresponding band appeared at the position of 250 bp and 125 bp respectively. Here, a 1 kb DNA ladder (TIANGEN Biotech Co., Ltd.) was employed in pannels of a-g, as the molecular size standard.

### 3.7. Blood (RBC, WBC, and % Hb), liver and kidney parameter check

Blood parameters were evaluated in all groups of mice, including the normal control group (Group I), control group (Group II), standard treatment group (Group III), and the compound-treated groups receiving compounds **1** and **2** (Group IV and Group V), respectively. (Table 3). After administration of standard drug and compounds **1** and **2,** hematological, hepatic and renal parameters were restored to near normal scale compared with those of the normal control group. There is dramatic change occurred like physiological worsening in the liver parameters such as elevation of ALT, AST and ALP in control group (Group II) compared to normal control group (Group I). Again, the compounds (**1** and **2**) attenuated the abnormality of hepatic function biomarkers (ALT, AST and ALP) in treatment groups with effects comparable to those of standard group. Besides, the bilirubin levels remain unchanged among treatment groups and control groups. Although, treatment group exerted a reduction in urea and creatinine levels but the change was not remarkable.

**Table 3:** Effect of compounds 1 and 2 on blood, liver, and kidney parameters in EAC cell bearing mice.

Treatment (mg/kg day^-1^)	Hematological test			Liver markers				Kidney markers	
	RBC x10^6^cell mm^-3^	WBC x10^6^ cell mm^-3^	% Hb gm/dL	ALT U/L	AST U/L	ALP U/L	Bilirubin mg/dL	Urea mg/dL	Creatinine mg/dL
Group-I (Normal control)	52.5 ± 1.1	5142.5 ± 7.2	12.11 ± 2.3	62	22	52	<10	20.4	0.8
Group-II (Diseased control)	23 ± 4.2	11456.2 ± 3.1	5.8 ± 1.3	99	53	161	<10	31.2	0.9
Group-III (Bleomycin 0.3 mg/kg	49.4 ± 3.3	6437.3 ± 2.1	10.1 ± 2.2	67	33	119	<10	23.3	0.9
Group-IV (Compound**1**, 5 mg/kg)	42.4 ± 1.5	7324.1 ± 5.2	8.9 ± 1.5	83	44	87	<10	27.3	0.8
Group-V (Compound **2**, 5 mg/kg)	46.3 ± 2.7	6287.6 ± 3.3	9.4 ± 3.1	76	39	60	<10	25.1	0.8

The results are shown in mean±SEM. Hematological parameters were examined in 5 groups of mice; among them Group III, IV and V were EAC cell bearing mice treated with bleomycin and compounds 1 and 2 for 5 days.

### 3.8. Molecular docking

Docking study was done to know the binding affinity as well possible potentiality of isolated compounds **1** and **2** with the selected genes P53, Bax, Bcl-2, Cyt-C and NF-kB. The lower binding energy was considered the higher binding affinity of the ligands. In the molecular docking study, a plenty number of hydrogen bond is responsible for binding the drugs with gene. The highest binding affinity is exhibited by both compounds **1** and **2** with Bcl-2 which are of −9.7 and −8.9 kcal/mol respectively (Table 4). Compound **1** and Bcl-2 were stabilized with nine conventional hydrogen bonds, three hydrophobic bonds and the proteins LYS22, ARG26, ASN22, ARG66, GLU119, VAL115, PHE71 were responsible for the binding energy (Fig 5 and **S2****-****S4 Table**). Compound **2** and Bcl-2 were equilibrated with seven conventional hydrogen bonds, five hydrophobic bonds. The proteins ASP61, LYS22, ARG26, SER64, ARG65, SER75, SER64, PHE63, LYS22, VAL115, VAL118 and VAL107 which were accountable to produce binding energy (Fig 6).

**Table 4:** Molecular docking scores retrieved from *silico* interactions of the drug candidates, the standard drug during the interaction with Bax (PDB ID: 4W63), Bcl-2 (PDB ID: 2W3L, Cyt-C (PDB ID: 2B4Z), and NF-κB (PDB ID: 1NFK) for assessing the anticancer activities.

Name of Compounds	PubChem CID	Binding affinity (kcal/mol)			
		Anticancer effects against various receptors			
		Bax	BcL-2	Cyt-C	NF-κB
		PDB ID: 4W63	PDB ID: 2W3L	PDB ID: 2B4Z	PDB ID: 1NFK
Compound **1**	21625636	-7.1	-9.7	-5.8	-5.3
Compound **2**	68315064	-7.4	-8.9	-6.4	-5.5
Bleomycin	5360373	-10.2	-9.3	-10.8	-9.5

**Fig 5:**
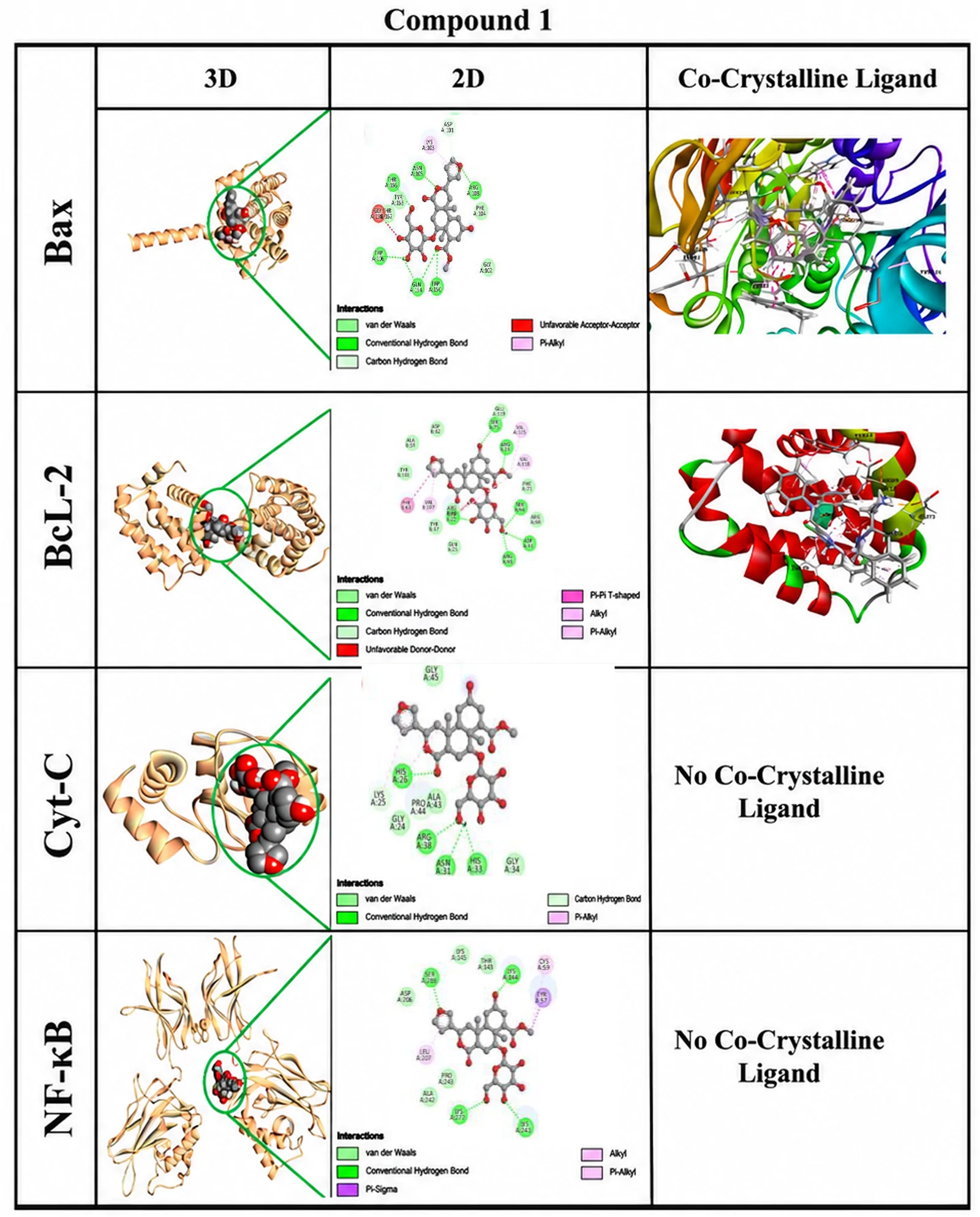
Representative three-dimensional and two-dimensional molecular docking interaction profiles of Compound 1 with Bax, Bcl-2, cytochrome c (Cyt-c), and NF-κB, with co-crystallized ligand binding sites.

**Fig 6:**
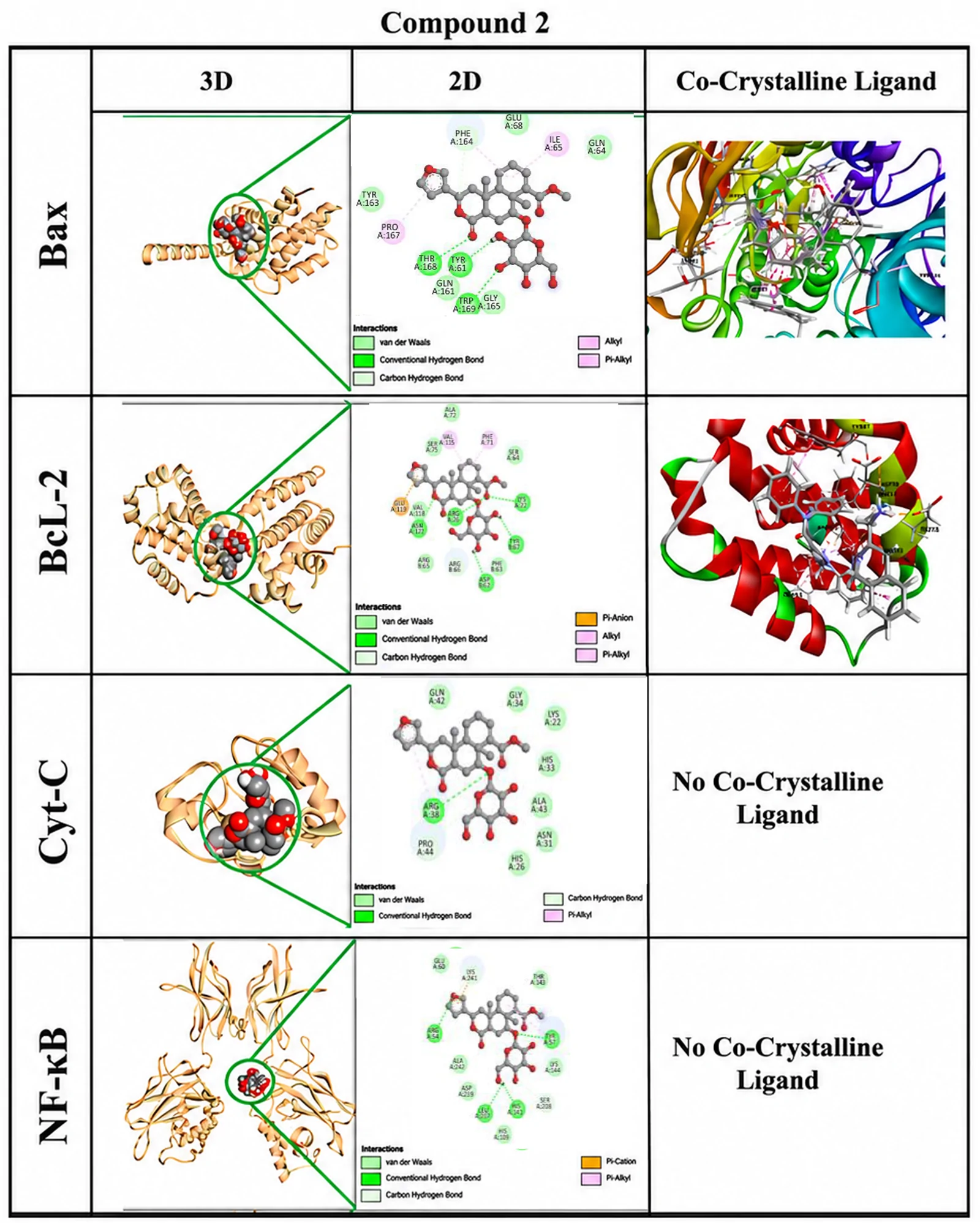
Representative three-dimensional and two-dimensional molecular docking interaction profiles of Compound 2 with Bax, Bcl-2, cytochrome c (Cyt-c), and NF-κB, with co-crystallized ligand binding sites.

Besides, compounds **1** and **2** demonstrated to have good binding interaction with Bax gene and the fitness score were −7.1 and −7.4 kcal/mol respectively (Table 4). The compound **1** and Bax gene were balanced with three hydrogen bonds and five hydrophobic bonds whereas compound **2** established approximately seven hydrogen bonds and two hydrophobic interactions (Fig 5). The proteins that were liable for binding with compound **1** were THR168, TRP169, TYR61, PHE164, ILE 65, PHE164, PRO167 while those interacting with compound **2** were ASN105, TRP106, ARG108, TRP150, GLN154, ASP101, LYS103 (S3 Table). Moreover, standard drug bleomycin has shown strongest binding affinity with all the genes (BAX, BcL-2, Cyt-C and NF-κB) which were −10.2, −9.3, −10.8 and −9.5 kcal/mol respectively (Table 4). The tested drugs 1 and 2 also revealed moderate interaction with Cyt C (−5.8 and −6.4 kcal/mol) and NF-κB (−5.3 and −5.5 kcal/mol) (Table 4) displaying in Fig 5 and Fig 6. To assess the binding sites and interaction patterns of compounds 1 and 2 with the selected proteins Bax and Bcl-2, the co-crystallized ligands available in the respective protein structures were used to identify the corresponding active binding sites representing in Fig 5 and Fig 6. The docking poses of compounds **1** and **2** were subsequently analyzed by comparing their interactions with the residues involved in ligand binding.

No co-crystallized ligands were available for cytochrome C (Cyt-C) and NF-κB. This indicate that experimentally determined ligand-binding complexes did not found in the selected proteins structures of Cyt C and NF-κB showing in Fig 5, Fig 6 and Fig 7.

**Fig 7:**
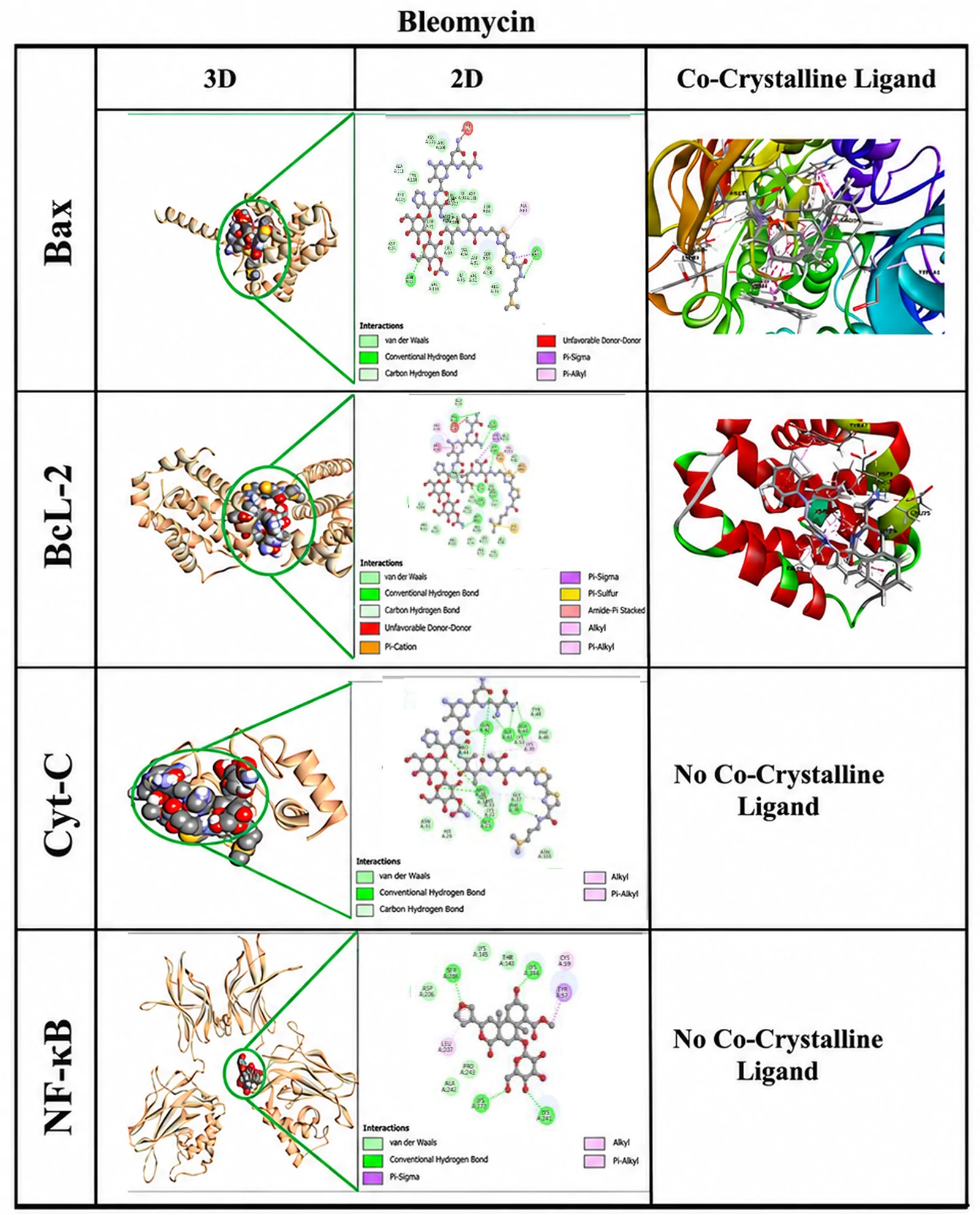
Representative three-dimensional and two-dimensional molecular docking interaction profiles of Bleomycin with Bax, Bcl-2, cytochrome c (Cyt-c), and NF-κB, with co-crystallized ligand binding sites.

## 4. Discussion

The prevalence of breast cancer continues to rise due to factors such as increased life expectancy, population growth, and the adoption of Western lifestyles, including high-fat diets, delayed marriage and childbirth, and reduced breastfeeding [45]. The leaves of *P. guajava* have been reported to contain bioactive compounds with potential anticancer properties [22]. In the present study, two diterpenoids, borapetoside F (**1**) and borapetoside B **(2**), were isolated from the EPG fraction, and this is the first report of their isolation (Fig 1) from this plant.

In the MTT viability assay, it has been observed that compound **1** demonstrated to have profound anticancer effect (83 μg/ml) compared to standard drug bleomycin (90.7 μg/ml) (Fig 2, and S1 Table).Besides, *in vivo* test, compound **1** showed greater cell growth inhibition (61.26%) compared to compound **2** (48.88%) relative to the control groups (Group II) (Table 2). Both compounds **1** and **2** are clerodane type diterpenoids, so that they manifested good anti-proliferative activity; notably, this is the first report for these two compounds from the *P. guajava* plant. Programmed cell death mechanism known as apoptosis characterized by distinct morphological change and activation of cell signaling pathway such as membrane blebbing, cell shrinkage, chromatin condensation, apoptotic body formation [46]. In fluorescence and optical microscopy study, the treatment groups accorded positive indication of apoptosis as compared to control group suggesting that the treatments may have induced programmed cell death. (Fig 3, S1 Table and S3 Fig). According to A. Wyllie, the cleavage of chromosomal DNA into small fragments is considered a hallmark feature of apoptosis [44].. In the DNA fragmentation assay, both compounds **1** and **2** as well as bleomycin gave smeared and splodge type band in 1% agarose gel electrophoresis which might be prime cause of cell death (Fig 4). Wang and his colleagues have shown protein known DNA fragmentation factor (DFF) which might have a great role in the initiation of apoptotic process [47]. DNA fragmentation factor (DFF) is essential in apoptosis, as it activates Ca^2+^/Mg^2+^ -dependent endonucleases responsible for cleaving chromosomal DNA during programmed cell death. Compounds **1** and **2**, identified as clerodane diterpenoids, may enhance the activation of these endonucleases, thereby triggering DNA fragmentation. These findings suggest that both compounds exert anti-proliferative activity by inducing apoptosis [48].. Previously, it has been reported that methanolic leaf extract of guava significantly suppresses the cell viability at a concentration of 0.57 mg/ml because it is likely attributed to the presence of bioactive phytoconstituents such as polyphenolic, terpenoid and flavonoid [49].

The cell cycle is regulated by multiple genes, and apoptosis is promoted through the upregulation of pro-apoptotic genes such as p53, Bax, and Cyt C, and the downregulation of anti-apoptotic genes including Bcl-2 and NF-κB [26]. In this study, compounds **1** and **2**, similar to bleomycin, increased the expression of p53, Bax, and Cyt C while reducing the expression of Bcl-2 and NF-κB in EAC cells (Fig 4). The tumor suppressor p53 induces apoptosis by facilitating DNA damage-mediated cell cycle arrest [50]. p53 helps control apoptosis by working with Bcl-2 family proteins. It activates Bax, which starts mitochondrial signals that lead to cell death. In contrast, the Bcl-2 protein prevents this process [51,52]. Moreover, Molecular docking study was also performed to validate resulting pharmacological effect and to explain the mechanistic pathway. Noncovalent hydrogen binding pattern in a biological system is thought to be facilitated drug protein binding [53]. Compound **1** and compound **2** appeared to exhibit favorable binding affinities toward the selected targets, including Bax, Cyt C, Bcl-2, and NF-κB. Compound **1** and **2** both are shown to have the highest binding affinity with Bcl-2 (−9.7 and −8.9 kcal/mol respectively) (Table 4) and deep binding pocket among selected genes. These results suggest that both compounds exhibit considerable structural stability and promising anticancer activity [54]. The second highest binding affinity was found with Bax gene of both compounds which are −7.1 and −7.4 kcal/mol respectively (Table 4). Bax promotes apoptosis by antagonizing Bcl-2, triggering mitochondrial cytochrome c release and subsequent activation of caspase-9 and caspase-3 [55]. The balance between p53-mediated pro-apoptotic and NF-κB-mediated anti-apoptotic signaling regulates tumor growth, cell survival, angiogenesis, and metastasis [56].. Compounds 1 and 2 appeared to exhibit apoptotic activity, which may, be partly associated with the downregulation of NF-κB (Fig 4). Though no co-crystalized ligand was acquired for Cyt C and NF-κB. It does not indicate that Cyt C and NF-κB can not bind with the compounds rather it is suggesting that the specific protein structures used in this study did not have a known ligand already attached to them when their structures were experimentally determined. Therefore, appropriate computational approaches based on the available structural features of the target proteins were used to determine exact docking sites for these proteins.

Earlier studies indicate that activating p53 can block NF-κB and increase ROS, thereby initiating the mitochondria-driven apoptotic pathway and leading to cell death [57].

Bone marrow depression and changes in liver and kidney function are common side effects of standard anticancer drugs. In this study, we measured blood (RBC, WBC, and hemoglobin percentage), liver (ALT, AST, ALP, and bilirubin), and kidney (urea and creatinine) parameters in mice with and without EAC cells. The control group (EAC-bearing mice without treatment) showed lower RBC and hemoglobin levels and higher WBC counts. In contrast, the groups treated with the compounds (Group IV and Group V) had values that returned close to normal (Table 3). There were no significant changes in liver bilirubin or kidney parameters in either the control or treated groups with EAC cells. The short duration of EAC cell exposure may not have been enough to cause noticeable changes in liver and kidney function. Earlier in the toxicity study, borapetoside B, C, and F (500 mg/kg/day) did not cause organ damage or dysfunction [58].

## 5. Conclusion

In conclusion, borapetoside F (**1**) and borapetoside B (**2**), isolated for the first time from the ethyl acetate fraction of *Psidium guajava* leaves, demonstrated promising anticancer activity against Ehrlich ascites carcinoma (EAC) cells. Their anticancer effects were associated with the induction of apoptosis, DNA fragmentation, and modulation of apoptosis-related gene expression, findings that were further supported by molecular docking analysis. Moreover, *in vivo* studies confirmed their therapeutic potential by significantly improving hematological and hepatic biochemical parameters in EAC-bearing mice. Collectively, these findings provide preliminary evidence that borapetoside F(**1**) and borapetoside B **(2**) are promising natural anticancer compounds. However, further mechanistic investigations, comprehensive toxicological evaluations, and preclinical studies are required to fully elucidate their molecular mechanisms of action and assess their therapeutic potential before clinical application.

## Supporting information

S1 Fig ^1^H-NMR and ^13^C-NMR spectrums of compound 1. (PDF)

S2 Fig ^1^H-NMR and ^13^C-NMR spectrums of compound 2. (PDF)

S3 Fig *In-vitro* cytotoxicity activity of control, standard and compounds 1 and 2 observed under an inverted light microscope. https://doi.org/10.1007/s11033-021-06136-z
(PDF)

S1 Table *In vitro* cytotoxic activity of compounds 1 and 2 from PG on EAC cell line. https://doi.org/10.1007/s11033-021-06136-z
(PDF)

S2 Table Molecular interactions of amino acids of the Bax protein (PDB ID: 5W60) with the isolated compounds, Borapetoside B, Borapetoside F and the standard drug bleomycin. https://doi.org/10.3390/ijms25052474
(PDF)

S3 Table Molecular interactions of amino acids of the Bcl-2 protein (PDB ID: 2W3L) with the isolated compounds, Borapetoside B, Borapetoside F and the standard drug bleomycin. https://doi.org/10.3390/ijms25052474
(PDF)

S4 Table Molecular interactions of amino acids of the Cyt-C protein (PDB ID: 2B4Z) with the isolated compounds, Borapetoside B, Borapetoside F and the standard drug bleomycin. https://doi.org/10.3390/ijms25052474
(PDF)

S5 Table Molecular interactions of amino acids of the NF-kB protein (PDB ID: 1NFK) with the isolated compounds, Borapetoside B, Borapetoside F and the standard drug bleomycin. https://doi.org/10.3390/ijms25052474
(PDF)

## References

[1] Dakappa SS, Adhikari R, Timilsina SS, Sajjekhan S. A review on the medicinal plant *Psidium Guajava* Linn. (myrtaceae). J Drug Delivery Ther. 2013;3(2). doi: 10.22270/jddt.v3i2.404

[2] Altemimi A, Lakhssassi N, Baharlouei A, Watson DG, Lightfoot DA. Phytochemicals: Extraction, isolation, and identification of bioactive compounds from plant extracts. Plants (Basel). 2017;6(4):42. doi: 10.3390/plants6040042 28937585 PMC5750618

[3] Gutiérrez-Grijalva EP, Picos-Salas MA, Leyva-López N, Criollo-Mendoza MS, Vazquez-Olivo G, Heredia JB. Flavonoids and phenolic acids from oregano: Occurrence, biological activity and health benefits. Plants (Basel). 2017;7(1):2. doi: 10.3390/plants7010002 29278371 PMC5874591

[4] Kumari KU, Rishitha G, Prasad KR, Suresh P. Value-added products of guava. Agric Update. 2017;12:2270–6. doi: 10.15740/HAS/AU/12.TECHSEAR(8)2017/2270-2276

[5] Karawya MS, Wahab SMA, Hifnawy MS, El-Gohary HM. Essential oil of Egyptian guava leaves. Egypt J Biomed Sci. 1999;209:217.

[6] Abdelrahim SI, Almagboul AZ, Omer MEA, Elegami A. Antimicrobial activity of *Psidium guajava* L. Fitoterapia. 2002;73(7–8):713–5. doi: 10.1016/s0367-326x(02)00243-5 12490238

[7] Begum S, Hassan SI, Ali SN, Siddiqui BS. Chemical constituents from the leaves of *Psidium guajava*. Nat Prod Res. 2004;18(2):135–40. doi: 10.1080/14786410310001608019 14984086

[8] Camarena-Tello JC, Martínez-Flores HE, Garnica-Romo MG, Padilla-Ramírez JS, Saavedra-Molina A, Alvarez-Cortes O, et al. Quantification of phenolic compounds and in vitro radical scavenging abilities with leaf extracts from two varieties of *Psidium guajava* L. Antioxidants (Basel). 2018;7(3):34. doi: 10.3390/antiox7030034 29495514 PMC5874520

[9] Venkatachalam RN, Singh K, Marar T. Phytochemical screening in vitro antioxidant activity of *psidium guajava*. Free Radicals and Antioxidants. 2012;2(1):31–6. doi: 10.5530/ax.2012.2.7

[10] Jamila M, Lobna A, Reham J. Total phenolic, flavonoid and antioxidant compounds of guava whey juice fortified by Moringa oleifera aqueous extract to extend shelf life. Int J Pharm Res Allied Sci. 2018;7(2):86–100. doi: 10.7717/peerj.4788

[11] Yaaser Q, Mustafa Z, Rashad M, Othman B, Waleed A, Hasan A. Assessment of antioxidant and antibacterial properties in two types of Yemeni guava cultivars. Biocatalysis and Agricultural Biotechnology. 2018;16:90–7. doi: 10.1016/j.bcab.2018.07.025

[12] Dos Santos WN, da Silva Sauthier MC, dos Santos AM, Santana DA, Azevedo RS, da Cruz Caldas J. Simultaneous determination of 13 phenolic bioactive compounds in guava (*Psidium guajava* L.) by HPLC-PAD with evaluation using PCA and neural network analysis. Microchem J. 2017;133:583–92. doi: 10.1089/glre.2016.201011

[13] Goławska S, Łukasik I, Chojnacki AA, Chrzanowski G. Flavonoids and Phenolic Acids Content in Cultivation and Wild Collection of European Cranberry Bush *Viburnum opulus* L. Molecules. 2023;28(5):2285. doi: 10.3390/molecules28052285 36903530 PMC10005456

[14] Yadav A, Tangpu T. Anticestodal efficacy of *Psidium guajava* against experimental Hymenolepis diminuta infection in rats. Indian J Pharmacol. 2006;38(1):29. doi: 10.4103/0253-7613.19849

[15] Nair R, Chanda S. In-vitro antimicrobial activity of *Psidium guajava* leaf extracts against clinically important pathogenic microbial strains. Braz J Microbiol. 2007;38:452–8. doi: 10.1590/S1517-83822007000300013

[16] Roy CK, Kamath JV, Asad M. Hepatoprotective activity of *Psidium guajava* Linn. leaf extract. Indian J Exp Biol. 2006;44(4):305–11. 16629373

[17] Chen H-Y, Yen G-C. Antioxidant activity and free radical-scavenging capacity of extracts from guava (*Psidium guajava* L.) leaves. Food Chemistry. 2007;101(2):686–94. doi: 10.1016/j.foodchem.2006.02.047

[18] Morales MA, Tortoriello J, Meckes M, Paz D, Lozoya X. Calcium-antagonist effect of quercetin and its relation with the spasmolytic properties of *Psidium guajava* L. Arch Med Res. 1994;25(1):17–21. 8019109

[19] Kaul TN, Middleton E Jr, Ogra PL. Antiviral effect of flavonoids on human viruses. J Med Virol. 1985;15(1):71–9. doi: 10.1002/jmv.1890150110 2981979

[20] Kaneuchi M, Sasaki M, Tanaka Y, Sakuragi N, Fujimoto S, Dahiya R. Quercetin regulates growth of Ishikawa cells through the suppression of EGF and cyclin D1. Int J Oncol. 2003;22(1):159–64. doi: 10.3892/ijo.22.1.159 12469199

[21] Newman DJ, Cragg GM. Natural Products as Sources of New Drugs over the Nearly Four Decades from 01/1981 to 09/2019. J Nat Prod. 2020;83(3):770–803. doi: 10.1021/acs.jnatprod.9b01285 32162523

[22] Lok B, Babu D, Tabana Y, Dahham SS, Adam MAA, Barakat K, et al. The anticancer potential of *Psidium guajava* (Guava) Extracts. Life (Basel). 2023;13(2):346. doi: 10.3390/life13020346 36836712 PMC9963020

[23] Sultana C, Kundo NK, Islam S, Ahmed R, Afrin S, Saqueeb N. Antioxidant, analgesic and antimicrobial activities of different fractions from methanolic extract of *Psidium guajava* L. leaves. Int J Pharm Sci Res. 2020;11:2733–8.

[24] Kupchan SM, Tsou G, Sigel CW. Datiscacin, a novel cytotoxic cucurbitacin 20-acetate from Datisca glomerata. J Org Chem. 1973;38(7):1420–1. doi: 10.1021/jo00947a041 4694234

[25] Khatun MCS, Muhit MA, Hossain MJ, Al-Mansur MA, Rahman SMA. Isolation of phytochemical constituents from Stevia rebaudiana (Bert.) and evaluation of their anticancer, antimicrobial and antioxidant properties via in vitro and in silico approaches. Heliyon. 2021;7(12):e08475. doi: 10.1016/j.heliyon.2021.e08475 34917793 PMC8645449

[26] Alam AK, Hossain AS, Khan MA, Kabir SR, Reza MA, Rahman MM, et al. The Antioxidative Fraction of White Mulberry Induces Apoptosis through Regulation of p53 and NFκB in EAC Cells. PLoS One. 2016;11(12):e0167536. doi: 10.1371/journal.pone.0167536 27936037 PMC5147903

[27] Khatun MCS, Muhit MA, Maruf MMH, Iqbal MA, Rahman SMA. Pharmacological evaluation of mangrove plant Rhizophora mucronata (Lam.) grown in the coastal area of Sundarbans. PLoS One. 2026;21(1):e0340646. doi: 10.1371/journal.pone.0340646 41575961 PMC12829777

[28] Asaduzzaman AKM, Hasan I, Chakrabortty A, Zaman S, Islam SS, Ahmed FRS, et al. Moringa oleifera seed lectin inhibits Ehrlich ascites carcinoma cell growth by inducing apoptosis through the regulation of Bak and NF-κB gene expression. Int J Biol Macromol. 2018;107(Pt B):1936–44. doi: 10.1016/j.ijbiomac.2017.10.070 29037871

[29] Kabir SR, Rahman MM, Amin R, Karim MR, Mahmud ZH, Hossain MT. Solanum tuberosum lectin inhibits Ehrlich ascites carcinoma cells growth by inducing apoptosis and G2/M cell cycle arrest. Tumour Biol. 2016;37(6):8437–44. doi: 10.1007/s13277-015-4735-x 26733170

[30] Karmakar PC, Yesmin R, Ali H, Rowshanul Habib M, Barman DN, Satter MA, et al. Antioxidant, cytotoxic and apoptotic potentials of seeds of Momordica subangulata subsp. renigera inhibit the growth of Ehrlich ascites carcinoma in mice. Food Measure. 2019;13(4):3049–59. doi: 10.1007/s11694-019-00227-z

[31] Kabir SR, Nabi MM, Haque A, Rokon Uz Zaman, Mahmud ZH, Reza MA. Pea lectin inhibits growth of Ehrlich ascites carcinoma cells by inducing apoptosis and G2/M cell cycle arrest in vivo in mice. Phytomedicine. 2013;20(14):1288–96. doi: 10.1016/j.phymed.2013.06.010 23867650

[32] Alam AHMK, Suzuki H, Tsukahara T. Retinoic acid treatment and cell aggregation independently regulate alternative splicing in P19 cells during neural differentiation. Cell Biol Int. 2010;34(6):631–43. doi: 10.1042/CBI20090332 20230377

[33] Khanam JA, Islam MF, Jesmin M, Ali MM. Antineoplastic activity of acetone semicarbazone (ASC) against Ehrlich ascites carcinoma (EAC) bearing mice. J Natn Sci Foundation Sri Lanka. 2010;38(4):225. doi: 10.4038/jnsfsr.v38i4.2649

[34] Berman HM, Battistuz T, Bhat TN, Bluhm WF, Bourne PE, Burkhardt K, et al. The Protein Data Bank. Acta Crystallogr D Biol Crystallogr. 2002;58(Pt 6 No 1):899–907. doi: 10.1107/s0907444902003451 12037327

[35] Lotfy K. Molecular modeling, docking and ADMET of dimethylthiohydantoin derivatives for prostate cancer treatment. J Biomed Sci Eng. 2015;6:91–117. doi: 10.4236/jbpc.2015.64010

[36] Mahmud S, Rafi MO, Paul GK, Promi MM, Shimu MSS, Biswas S, et al. Designing a multi-epitope vaccine candidate to combat MERS-CoV by employing an immunoinformatics approach. Sci Rep. 2021;11(1):15431. doi: 10.1038/s41598-021-92176-1 34326355 PMC8322212

[37] Bikadi Z, Hazai E. Application of the PM6 semi-empirical method to modeling proteins enhances docking accuracy of AutoDock. J Cheminform. 2009;1:15. doi: 10.1186/1758-2946-1-15 20150996 PMC2820493

[38] Trott O, Olson AJ. AutoDock Vina: Improving the speed and accuracy of docking with a new scoring function, efficient optimization, and multithreading. J Comput Chem. 2010;31(2):455–61. doi: 10.1002/jcc.21334 19499576 PMC3041641

[39] Tian W, Chen C, Lei X, Zhao J, Liang J. CASTp 3.0: computed atlas of surface topography of proteins. Nucleic Acids Res. 2018;46(W1):W363–7. doi: 10.1093/nar/gky473 29860391 PMC6031066

[40] Systèmes D, Biovia. Discovery Studio Visualizer. San Diego: Dassault Systèmes. 2017.

[41] Akhila A, Rani K, Thakur RS. Biosynthesis of the clerodane furano-diterpene lactone skeleton in Tinospora cordifolia. Phytochemistry. 1991;30(8):2573–6. doi: 10.1016/0031-9422(91)85103-7

[42] Fukuda N, Yonemitsu M, Kimura T. Studies on the Constituents of the Stems of Tinospora tuberculata, IV. Isolation and Structure Elucidation of the Five New Furanoid Diterpene Glycosides Borapetoside C–G. Liebigs Ann Chem. 1993;1993(5):491–5. doi: 10.1002/jlac.199319930181

[43] Martin TS, Ohtani K, Kasai R, Yamasaki K. Furanoid diterpene glucosides from Tinospora rumphii. Phytochemistry. 1996;42(1):153–8. doi: 10.1016/0031-9422(95)00902-7 8728064

[44] Wyllie AH. Apoptosis (the 1992 Frank Rose Memorial Lecture). Br J Cancer. 1993;67:205–8. doi: 10.1038/bjc.1993.40 PMC1968187 8431353

[45] Shamsi T. Burden of breast cancer in Bangladesh-current and future and financing treatment with link to willingness to pay. Int J Community Med Public Health. 2021;8(11):5525. doi: 10.18203/2394-6040.ijcmph20214295

[46] Shimu AS, Miah M, Billah M, Karmakar S, Mohanto SC, Khatun R, et al. A comparative study of biological potentiality and EAC cell growth inhibition activity of Phyllanthus acidus (L.) fruit pulp and seed in Bangladesh. Saudi J Biol Sci. 2021;28(3):2014–22. doi: 10.1016/j.sjbs.2021.01.004 33732088 PMC7938185

[47] Wang H-Y, Wang H, Wang J-H, Wang Q, Ma Q-F, Chen Y-Y. Protocatechuic Acid Inhibits Inflammatory Responses in LPS-Stimulated BV2 Microglia via NF-κB and MAPKs Signaling Pathways. Neurochem Res. 2015;40(8):1655–60. doi: 10.1007/s11064-015-1646-6 26134310

[48] Islam F, Khatun H, Khatun M, Ali SMM, Khanam JA. Growth inhibition and apoptosis of Ehrlich ascites carcinoma cells by the methanol extract of Eucalyptus camaldulensis. Pharm Biol. 2014;52(3):281–90. doi: 10.3109/13880209.2013.834365 24102623

[49] Peng C-C, Peng C-H, Chen K-C, Hsieh C-L, Peng RY. The Aqueous Soluble Polyphenolic Fraction of *Psidium guajava* Leaves Exhibits Potent Anti-Angiogenesis and Anti-Migration Actions on DU145 Cells. Evid Based Complement Alternat Med. 2011;2011:219069. doi: 10.1093/ecam/neq005 21799674 PMC3135903

[50] Nakano K, Vousden KH. PUMA, a novel proapoptotic gene, is induced by p53. Mol Cell. 2001;7(3):683–94. doi: 10.1016/s1097-2765(01)00214-3 11463392

[51] Yu J, Zhang L. PUMA, a potent killer with or without p53. Oncogene. 2008;27 Suppl 1(Suppl 1):S71-83. doi: 10.1038/onc.2009.45 19641508 PMC2860432

[52] Yang J, Liu X, Bhalla K, Kim CN, Ibrado AM, Cai J, et al. Prevention of apoptosis by Bcl-2: release of cytochrome c from mitochondria blocked. Science. 1997;275(5303):1129–32. doi: 10.1126/science.275.5303.1129 9027314

[53] Chen D, Oezguen N, Urvil P, Ferguson C, Dann SM, Savidge TC. Regulation of protein-ligand binding affinity by hydrogen bond pairing. Sci Adv. 2016;2(3):e1501240. doi: 10.1126/sciadv.1501240 27051863 PMC4820369

[54] Arthur DE, Uzairu A. Molecular docking studies on the interaction of NCI anticancer analogues with human Phosphatidylinositol 4,5-bisphosphate 3-kinase catalytic subunit. Journal of King Saud University - Science. 2019;31(4):1151–66. doi: 10.1016/j.jksus.2019.01.011

[55] Guo B, Zhai D, Cabezas E, Welsh K, Nouraini S, Satterthwait AC, et al. Humanin peptide suppresses apoptosis by interfering with Bax activation. Nature. 2003;423(6938):456–61. doi: 10.1038/nature01627 12732850

[56] Karin M, Cao Y, Greten FR, Li Z-W. NF-kappaB in cancer: from innocent bystander to major culprit. Nat Rev Cancer. 2002;2(4):301–10. doi: 10.1038/nrc780 12001991

[57] Kannan K, Jain S. Oxidative stress and apoptosis. Pathophysiology. 2000;7(3):153–63. doi: 10.1016/s0928-4680(00)00053-5 10996508

[58] Kabir SR, Nabi MM, Nurujjaman M, Abu Reza M, Alam AHMK, Uz Zaman R, et al. Momordica charantia seed lectin: toxicity, bacterial agglutination and antitumor properties. Appl Biochem Biotechnol. 2015;175(5):2616–28. doi: 10.1007/s12010-014-1449-2 25542240

