# Supplementary material for: Integrated In vitro and in vivo evaluation of anticancer potentials of two clerodane diterpenes isolated from Psidium guajava (L.) through molecular docking approaches
Source: PLoS One. 2026 Nov 13;21(11):e0362655. doi: 10.1371/journal.pone.0362655 (PMC13766287; doi:10.1371/journal.pone.0362655)
Supplement: Supplementary file 1 [file pone.0362655.s001.pdf]

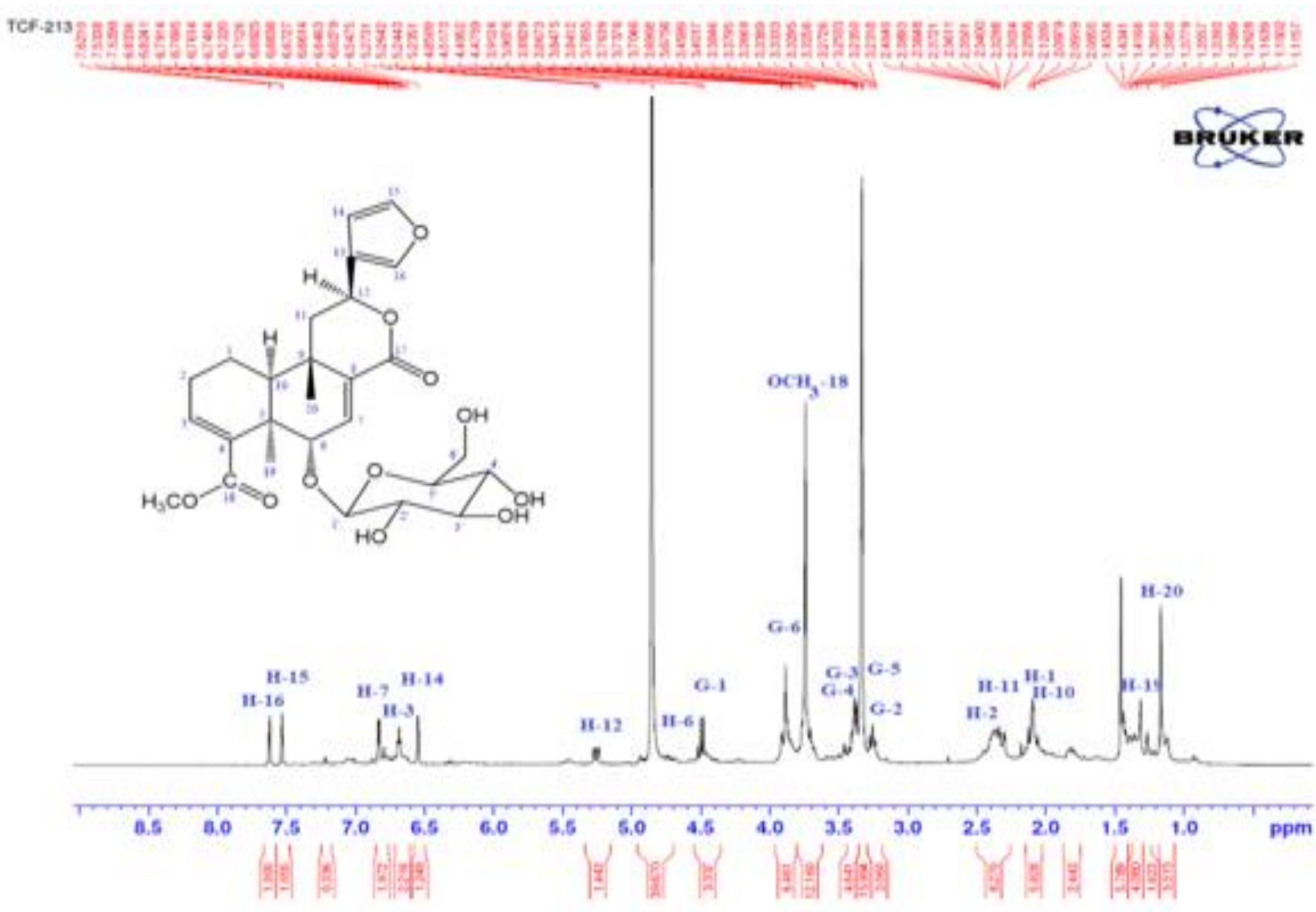

TCF-213
OCH3-18
G-6
G-3
G-5
G-4
G-2
G-1
H-15
H-16
H-7
H-14
H-3
H-12
H-6
H-11
H-1
H-10
H-2
H-19
H-20
8.5
8.0
7.5
7.0
6.5
6.0
5.5
5.0
4.5
4.0
3.5
3.0
2.5
2.0
1.5
1.0
ppm

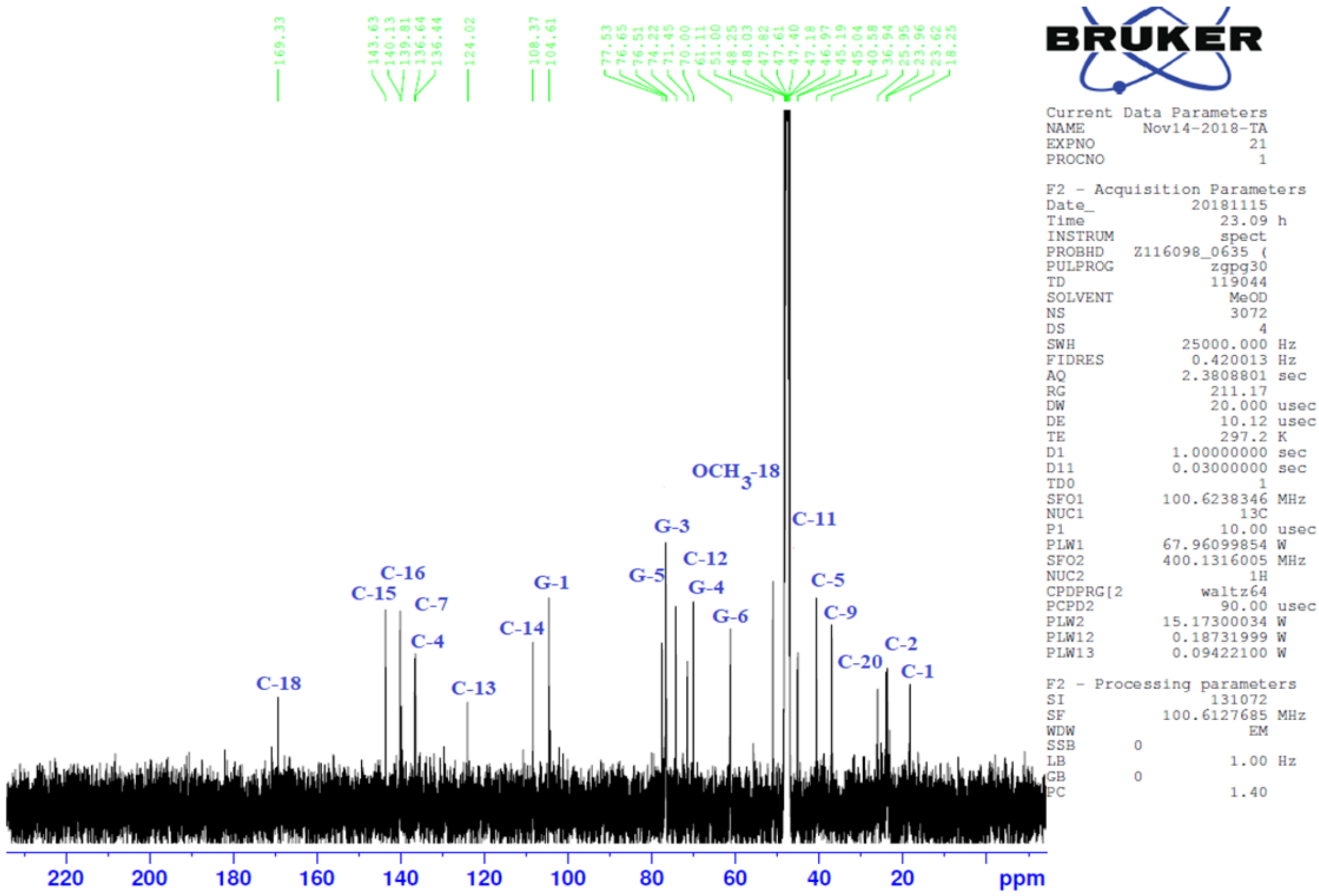

169.33
143.63
140.13
139.81
136.64
136.44
124.02
108.37
104.61
77.53
76.65
76.51
74.22
71.45
70.00
61.11
51.00
48.25
48.03
47.82
47.61
47.40
47.18
46.97
45.19
45.04
40.58
36.94
25.95
23.96
23.62
18.25
BRUKER
Current Data Parameters
NAME Nov14-2018-TA
EXPNO 21
PROCNO 1
F2 - Acquisition Parameters
Date_ 20181115
Time 23.09 h
INSTRUM spect
PROBHD Z116098_0635 (
PULPROG zgpg30
TD 119044
SOLVENT MeOD
NS 3072
DS 4
SWH 25000.000 Hz
FIDRES 0.420013 Hz
AQ 2.3808801 sec
RG 211.17
DW 20.000 usec
DE 10.12 usec
TE 297.2 K
D1 1.00000000 sec
D11 0.03000000 sec
TD0 1
SFO1 100.6238346 MHz
NUC1 13C
P1 10.00 usec
PLW1 67.96099854 W
SFO2 400.1316005 MHz
NUC2 1H
CPDPRG[2 waltz64
PCPD2 90.00 usec
PLW2 15.17300034 W
PLW12 0.18731999 W
PLW13 0.09422100 W
F2 - Processing parameters
SI 131072
SF 100.6127685 MHz
WDW EM
SSB 0
LB 1.00 Hz
GB 0
PC 1.40
OCH3-18
C-11
G-3
C-12
G-5
G-4
C-5
C-16
C-15
C-7
G-1
C-9
G-6
C-14
C-4
C-2
C-20
C-1
C-18
C-13
220
200
180
160
140
120
100
80
60
40
20
ppm
