# Supplementary material for: Integrated In vitro and in vivo evaluation of anticancer potentials of two clerodane diterpenes isolated from Psidium guajava (L.) through molecular docking approaches
Source: PLoS One. 2026 Nov 13;21(11):e0362655. doi: 10.1371/journal.pone.0362655 (PMC13766287; doi:10.1371/journal.pone.0362655)
Supplement: Supplementary file 2 [file pone.0362655.s002.pdf]

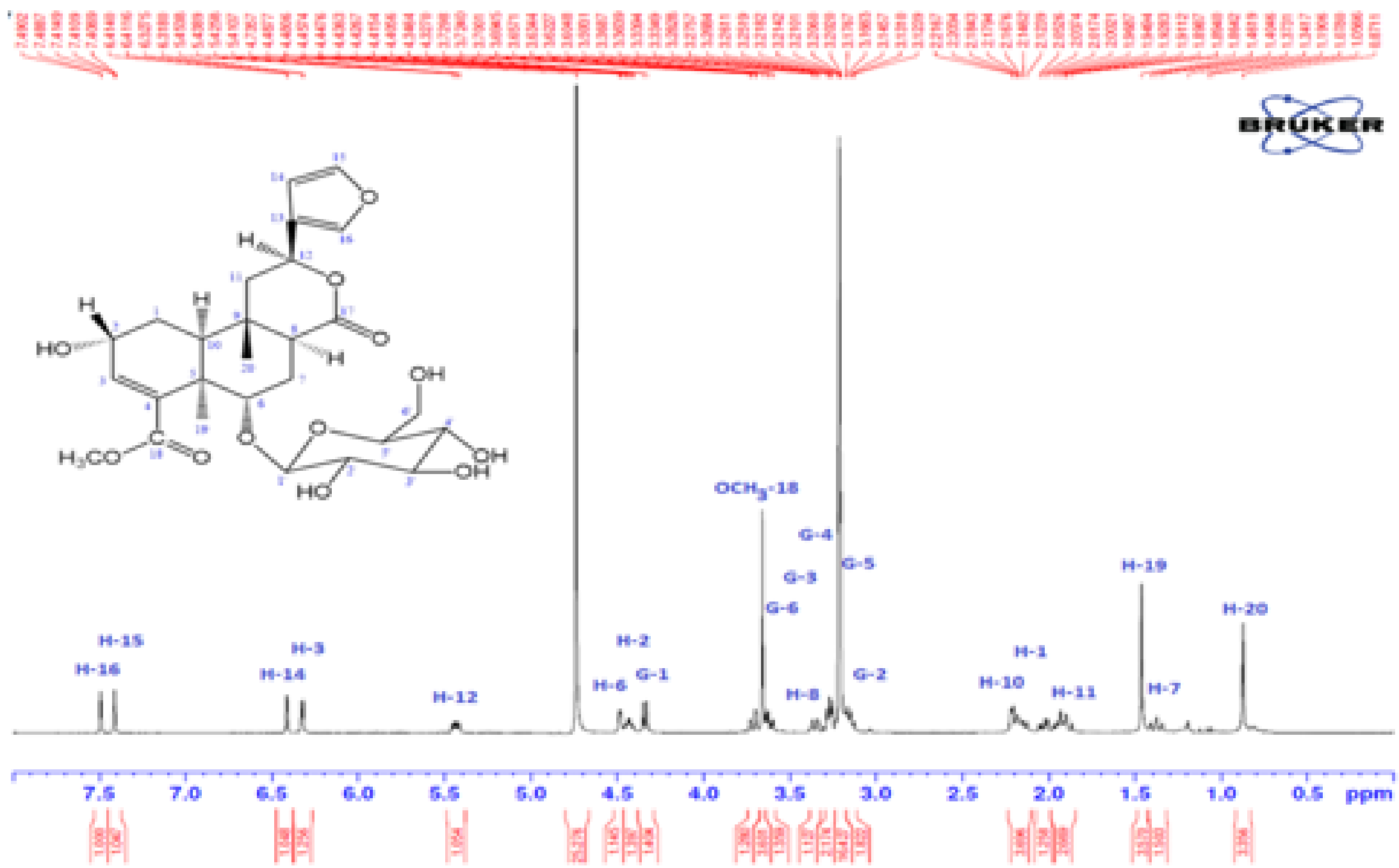
BRUKER
OCH3-18
G-4
G-5
G-3
G-6
H-19
H-20
H-15
H-16
H-3
H-14
H-2
G-1
H-6
H-12
H-8
G-2
H-1
H-10
H-11
H-7
7.5
7.0
6.5
6.0
5.5
5.0
4.5
4.0
3.5
3.0
2.5
2.0
1.5
1.0
0.5
ppm

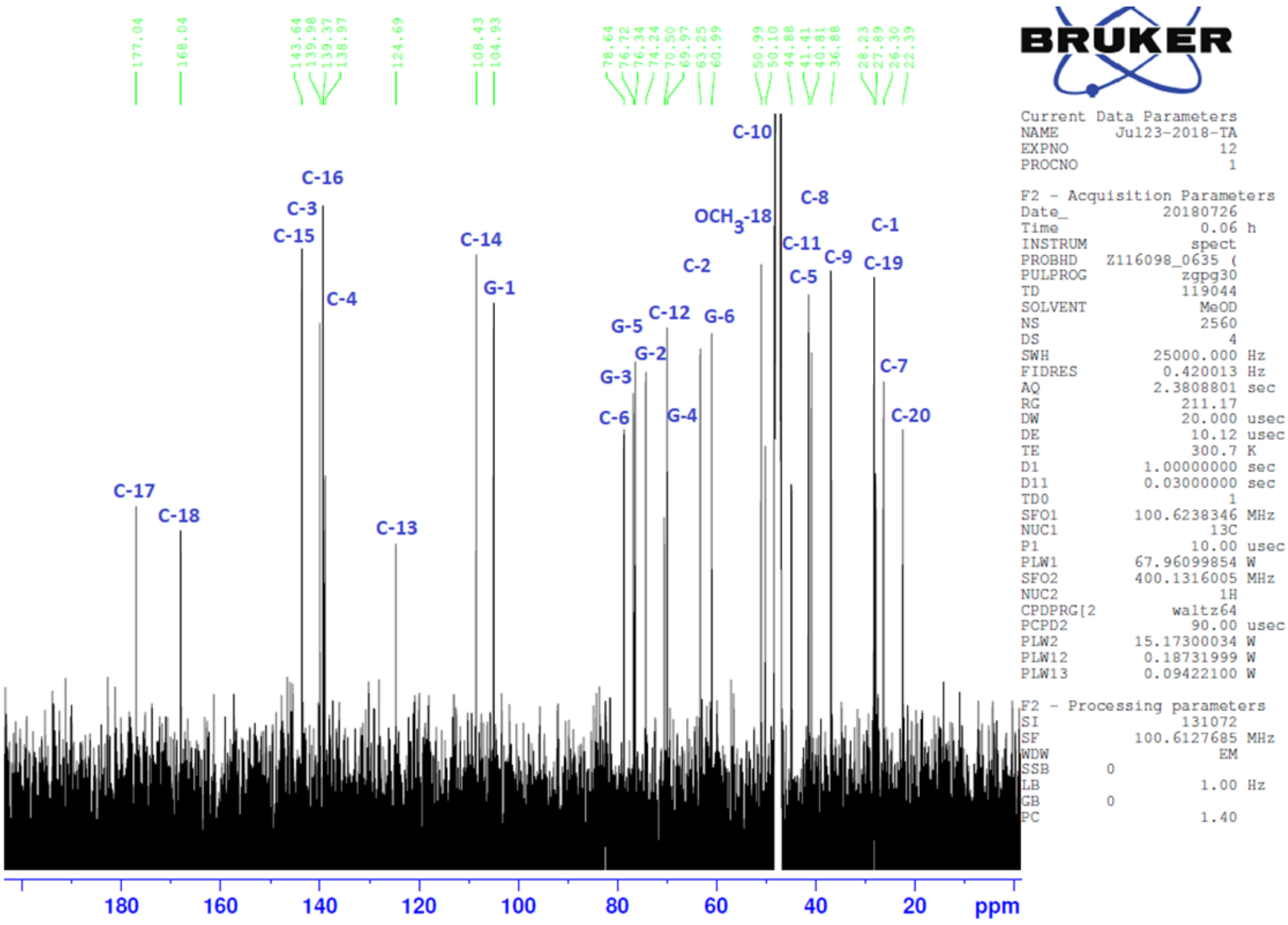

177.04
168.04
143.64
139.98
139.37
138.97
124.69
108.43
104.93
78.64
76.72
76.34
74.24
70.50
69.97
63.25
60.99
50.99
50.10
44.88
41.41
40.81
36.88
28.23
27.89
26.30
22.39
C-10
C-16
C-3
C-15
C-4
C-14
G-1
OCH3-18
C-8
C-11
C-5
C-9
C-1
C-19
C-2
C-12
G-6
G-5
G-2
G-3
G-4
C-6
C-7
C-20
C-17
C-18
C-13
180
160
140
120
100
80
60
40
20
ppm
BRUKER
Current Data Parameters
NAME Jul23-2018-TA
EXPNO 12
PROCNO 1
F2 - Acquisition Parameters
Date_ 20180726
Time 0.06 h
INSTRUM spect
PROBHD Z116098_0635 (
PULPROG zgpg30
TD 119044
SOLVENT MeOD
NS 2560
DS 4
SWH 25000.000 Hz
FIDRES 0.420013 Hz
AQ 2.3808801 sec
RG 211.17
DW 20.000 usec
DE 10.12 usec
TE 300.7 K
D1 1.00000000 sec
D11 0.03000000 sec
TD0 1
SFO1 100.6238346 MHz
NUC1 13C
P1 10.00 usec
PLW1 67.96099854 W
SFO2 400.1316005 MHz
NUC2 1H
CPDPRG[2 waltz64
PCPD2 90.00 usec
PLW2 15.17300034 W
PLW12 0.18731999 W
PLW13 0.09422100 W
F2 - Processing parameters
SI 131072
SF 100.6127685 MHz
WDW EM
SSB 0
LB 1.00 Hz
GB 0
PC 1.40
