# Supplementary material for: Integrated In vitro and in vivo evaluation of anticancer potentials of two clerodane diterpenes isolated from Psidium guajava (L.) through molecular docking approaches
Source: PLoS One. 2026 Nov 13;21(11):e0362655. doi: 10.1371/journal.pone.0362655 (PMC13766287; doi:10.1371/journal.pone.0362655)
Supplement: Supplementary file 4 [file pone.0362655.s004.pdf]

Compound	Concentration (μg/ml)	Growth inhibition of EAC cells (%)	IC_{50} value (μg/ml)
Control (DMSO)	2.5	--	--
Bleomycin	62.5	43.7	
	125	70.4	31.3
	250	90.7	
	500	94.8	
**1**	62.5	33.7	83.0
	125	58.7	
	250	62.5	
	500	82.4	
**2**	62.5	46.3	136.1
	125	55.5	
	250	62.3	
	500	79.8	
