# Supplementary material for: Integrated In vitro and in vivo evaluation of anticancer potentials of two clerodane diterpenes isolated from Psidium guajava (L.) through molecular docking approaches
Source: PLoS One. 2026 Nov 13;21(11):e0362655. doi: 10.1371/journal.pone.0362655 (PMC13766287; doi:10.1371/journal.pone.0362655)
Supplement: Supplementary file 5 [file pone.0362655.s005.pdf]

Compound	Bond (AA and Ligand)	Bond length	Bond type	Bond nature
Borapetoside F	A:THR168:HG1 - N:UNK1:O	2.37359	Hydrogen Bond	Conventional Hydrogen Bond
	A:TRP169:HE1 - N:UNK1:O	2.56027	Hydrogen Bond	Conventional Hydrogen Bond
	N:UNK1:H - A:TYR61:OH	1.90665	Hydrogen Bond	Conventional Hydrogen Bond
	N:UNK1:C - A:PHE164:O	2.93833	Hydrogen Bond	Carbon Hydrogen Bond
	N:UNK1:C - N:UNK1:O	3.72628	Hydrogen Bond	Carbon Hydrogen Bond
	A:ILE65 - N:UNK1	4.31724	Hydrophobic	Alkyl
	A:PHE164 - N:UNK1	5.41573	Hydrophobic	Pi-Alkyl
	N:UNK1 - A:PRO167	4.44384	Hydrophobic	Pi-Alkyl
Borapetoside B	A:ASN105:HD22 - N:UNK1:O	2.61412	Hydrogen Bond	Conventional Hydrogen Bond
	A:TRP106:HE1 - N:UNK1:O	2.96382	Hydrogen Bond	Conventional Hydrogen Bond
	A:ARG108:HH22 - N:UNK1:O	2.39021	Hydrogen Bond	Conventional Hydrogen Bond
	A:TRP150:HE1 - N:UNK1:O	2.2149	Hydrogen Bond	Conventional Hydrogen Bond
	A:GLN154:HE21 - N:UNK1:O	2.52624	Hydrogen Bond	Conventional Hydrogen Bond
	A:THR166:HG1 - N:UNK1:O	2.63471	Hydrogen Bond	Conventional Hydrogen Bond
	N:UNK1:H - A:GLN154:OE1	2.81093	Hydrogen Bond	Conventional Hydrogen Bond
	N:UNK1:C - A:ASP101:OD2	3.68085	Hydrogen Bond	Carbon Hydrogen Bond
	N:UNK1 - A:LYS103	4.44788	Hydrophobic	Pi-Alkyl
Bleomycin	N:UNK1:H - N:UNK1:O	2.52677	Hydrogen Bond	Conventional Hydrogen Bond
	N:UNK1:H - N:UNK1:O	2.3507	Hydrogen Bond	Conventional Hydrogen Bond
	N:UNK1:H - N:UNK1:O	2.31799	Hydrogen Bond	Conventional Hydrogen Bond
	N:UNK1:H - N:UNK1:O	2.9257	Hydrogen Bond	Conventional Hydrogen Bond
	N:UNK1:H - N:UNK1:S	2.69682	Hydrogen Bond	Conventional Hydrogen Bond
	N:UNK1:H - A:LYS93:O	2.47215	Hydrogen Bond	Conventional Hydrogen Bond
	N:UNK1:HN - N:UNK1:O	2.85853	Hydrogen Bond	Conventional Hydrogen Bond
	N:UNK1:HN - N:UNK1:O	2.49131	Hydrogen Bond	Conventional Hydrogen Bond
	N:UNK1:C - A:ALA111:O	3.60171	Hydrogen Bond	Carbon Hydrogen Bond
	A:LYS93:CB - N:UNK1	3.90144	Hydrophobic	Pi-Sigma
	N:UNK1 - A:ALA83	4.98747	Hydrophobic	Pi-Alkyl
