# Supplementary material for: Integrated In vitro and in vivo evaluation of anticancer potentials of two clerodane diterpenes isolated from Psidium guajava (L.) through molecular docking approaches
Source: PLoS One. 2026 Nov 13;21(11):e0362655. doi: 10.1371/journal.pone.0362655 (PMC13766287; doi:10.1371/journal.pone.0362655)
Supplement: Supplementary file 6 [file pone.0362655.s006.pdf]

Compound	Bond (AA and Ligand)	Bond length	Bond type	Bond nature
Borapetoside F	A:LYS22:HZ3 - N:UNK1:O	2.60287	Hydrogen Bond	Conventional Hydrogen Bond
	A:ARG26:HE - N:UNK1:O	2.76684	Hydrogen Bond	Conventional Hydrogen Bond
	A:ARG26:HH11 - N:UNK1:O	2.14606	Hydrogen Bond	Conventional Hydrogen Bond
	A:ARG26:HH12 - N:UNK1:O	2.23728	Hydrogen Bond	Conventional Hydrogen Bond
	A:ASN122:HD21 - N:UNK1:O	2.73393	Hydrogen Bond	Conventional Hydrogen Bond
	A:ASN122:HD22 - N:UNK1:O	2.86754	Hydrogen Bond	Conventional Hydrogen Bond
	N:UNK1:H - B:ASP62:O	2.75311	Hydrogen Bond	Conventional Hydrogen Bond
	N:UNK1:H - B:TYR67:OH	2.04803	Hydrogen Bond	Conventional Hydrogen Bond
	B:ARG66:CD - N:UNK1:O	3.24379	Hydrogen Bond	Carbon Hydrogen Bond
	A:GLU119:OE2 - N:UNK1	3.73261	Electrostatic	Pi-Anion
	A:VAL115 - N:UNK1	5.11384	Hydrophobic	Alkyl
	A:PHE71 - N:UNK1	5.45442	Hydrophobic	Pi-Alkyl
Borapetoside B	N:UNK1:H - A:ASP61:O	2.59313	Hydrogen Bond	Conventional Hydrogen Bond
	A:LYS22:HZ1 - N:UNK1:O	2.10598	Hydrogen Bond	Conventional Hydrogen Bond
	A:ARG26:HH11 - N:UNK1:O	2.45155	Hydrogen Bond	Conventional Hydrogen Bond
	A:SER64:HG - N:UNK1:O	2.88076	Hydrogen Bond	Conventional Hydrogen Bond
	A:ARG65:HN - N:UNK1:O	2.51536	Hydrogen Bond	Conventional Hydrogen Bond
	A:SER75:HG - N:UNK1:O	2.37756	Hydrogen Bond	Conventional Hydrogen Bond
	N:UNK1:C - A:SER64:OG	3.06783	Hydrogen Bond	Carbon Hydrogen Bond
	N:UNK1 - B:PHE63	5.33269	Hydrophobic	Pi-Pi T-shaped
	N:UNK1:C - A:LYS22	4.8719	Hydrophobic	Alkyl
	N:UNK1:C - A:VAL115	4.48205	Hydrophobic	Alkyl
	N:UNK1:C - A:VAL118	3.52886	Hydrophobic	Alkyl
	N:UNK1 - B:VAL107	5.20567	Hydrophobic	Pi-Alkyl
Bleomycin	A:ARG26:HH11 - N:UNK1:O	1.97512	Hydrogen Bond	Conventional Hydrogen Bond
	A:ARG26:HH21 - N:UNK1:O	2.73312	Hydrogen Bond	Conventional Hydrogen Bond
	A:SER75:HG - N:UNK1:O	1.73439	Hydrogen Bond	Conventional Hydrogen Bond
	N:UNK1:H - N:UNK1:O	2.07767	Hydrogen Bond	Conventional Hydrogen Bond
	N:UNK1:H - A:ALA72:O	3.02946	Hydrogen Bond	Conventional Hydrogen Bond
	N:UNK1:H - N:UNK1:O	2.38993	Hydrogen Bond	Conventional Hydrogen Bond
	N:UNK1:H - B:LEU160:O	2.79181	Hydrogen Bond	Conventional Hydrogen Bond
	N:UNK1:H - B:ASP62:OD2	2.52426	Hydrogen Bond	Conventional Hydrogen Bond
	N:UNK1:H - N:UNK1:O	2.30325	Hydrogen Bond	Conventional Hydrogen Bond
	N:UNK1:H - N:UNK1:O	1.83253	Hydrogen Bond	Conventional Hydrogen Bond
	N:UNK1:H - A:GLU119:OE2	2.31207	Hydrogen Bond	Conventional Hydrogen Bond
	N:UNK1:H - A:SER64:OG	2.25638	Hydrogen Bond	Conventional Hydrogen Bond
	N:UNK1:HN - N:UNK1:O	1.95115	Hydrogen Bond	Conventional Hydrogen Bond
	N:UNK1:C - A:ALA72:O	3.41455	Hydrogen Bond	Carbon Hydrogen Bond
	A:ARG65:NH1 - N:UNK1	4.01067	Electrostatic	Pi-Cation
	B:ARG66:NH2 - N:UNK1	4.25552	Electrostatic	Pi-Cation

	N:UNK1:C - A:PHE71	3.70464	Hydrophobic	Pi-Sigma
	N:UNK1:S - B:PHE63	5.06317	Other	Pi-Sulfur
	A:ARG68:C,O;ARG69:N - N:UNK1	5.33912	Hydrophobic	Amide-Pi Stacked
	N:UNK1:C - A:ARG68	3.74663	Hydrophobic	Alkyl
	N:UNK1:C - A:ARG69	4.29047	Hydrophobic	Alkyl
	N:UNK1:C - A:VAL115	4.49153	Hydrophobic	Alkyl
	N:UNK1 - A:ARG69	4.79562	Hydrophobic	Pi-Alkyl
	N:UNK1 - A:ALA72	4.16986	Hydrophobic	Pi-Alkyl
