# Supplementary material for: Integrated In vitro and in vivo evaluation of anticancer potentials of two clerodane diterpenes isolated from Psidium guajava (L.) through molecular docking approaches
Source: PLoS One. 2026 Nov 13;21(11):e0362655. doi: 10.1371/journal.pone.0362655 (PMC13766287; doi:10.1371/journal.pone.0362655)
Supplement: Supplementary file 7 [file pone.0362655.s007.pdf]

Compound	Bond (AA and Ligand)	Bond length	Bond type	Bond nature
Borapetoside F	A:HIS26:HE2 - A:PRO44:O	1.87687	Hydrogen Bond	Conventional Hydrogen Bond
	A:ARG38:HN - A:LEU35:O	2.14144	Hydrogen Bond	Conventional Hydrogen Bond
	A:ARG38:HH11 - A:HIS33:O	2.20679	Hydrogen Bond	Conventional Hydrogen Bond
	A:ARG38:HH11 - N:UNK1:O	2.70172	Hydrogen Bond	Conventional Hydrogen Bond
	A:ARG38:HH21 - A:HIS33:O	2.40883	Hydrogen Bond	Conventional Hydrogen Bond
	A:TRP59:HN - A:ARG38:O	1.84543	Hydrogen Bond	Conventional Hydrogen Bond
	A:PRO44:CD - N:UNK1:O	3.20458	Hydrogen Bond	Carbon Hydrogen Bond
	A:TRP59:CD1 - A:ARG38:O	3.59394	Hydrogen Bond	Carbon Hydrogen Bond
	N:UNK1 - A:ARG38	5.16682	Hydrophobic	Pi-Alkyl
Borapetoside B	N:UNK1:H - N:UNK1:O	2.22655	Hydrogen Bond	Conventional Hydrogen Bond
	N:UNK1:H - A:ASN31:OD1	2.59652	Hydrogen Bond	Conventional Hydrogen Bond
	N:UNK1:H - A:HIS33:O	2.27809	Hydrogen Bond	Conventional Hydrogen Bond
	N:UNK1:H - N:UNK1:O	1.85954	Hydrogen Bond	Conventional Hydrogen Bond
	A:HIS26:HN - N:UNK1:O	2.68588	Hydrogen Bond	Conventional Hydrogen Bond
	A:ARG38:HH21 - N:UNK1:O	2.2974	Hydrogen Bond	Conventional Hydrogen Bond
	A:LYS25:CA - N:UNK1:O	3.57481	Hydrogen Bond	Carbon Hydrogen Bond
	A:HIS33:CD2 - N:UNK1:O	3.61278	Hydrogen Bond	Carbon Hydrogen Bond
	A:PRO44:CD - N:UNK1:O	3.65072	Hydrogen Bond	Carbon Hydrogen Bond
	N:UNK1 - A:LYS25	5.41222	Hydrophobic	Pi-Alkyl
Bleomycin	A:HIS26 - N:UNK1:C	4.60679	Hydrophobic	Pi-Alkyl
	N:UNK1:H - A:GLY41:O	2.38745	Hydrogen Bond	Conventional Hydrogen Bond
	N:UNK1:H - A:ALA43:O	2.09323	Hydrogen Bond	Conventional Hydrogen Bond
	N:UNK1:H - A:GLY41:O	2.75351	Hydrogen Bond	Conventional Hydrogen Bond
	N:UNK1:H - N:UNK1:O	2.7914	Hydrogen Bond	Conventional Hydrogen Bond
	N:UNK1:H - N:UNK1:O	2.73245	Hydrogen Bond	Conventional Hydrogen Bond
	N:UNK1:H - N:UNK1:O	2.24718	Hydrogen Bond	Conventional Hydrogen Bond
	N:UNK1:H - N:UNK1:O	2.70237	Hydrogen Bond	Conventional Hydrogen Bond
	N:UNK1:H - N:UNK1:O	2.64654	Hydrogen Bond	Conventional Hydrogen Bond
	N:UNK1:H - A:PHE36:O	1.97456	Hydrogen Bond	Conventional Hydrogen Bond
	N:UNK1:HN - N:UNK1:O	2.59127	Hydrogen Bond	Conventional Hydrogen Bond
	A:GLY23:HN - N:UNK1:O	2.44563	Hydrogen Bond	Conventional Hydrogen Bond
	A:ARG38:HH11 - N:UNK1:O	2.63464	Hydrogen Bond	Conventional Hydrogen Bond
	A:ARG38:HH12 - N:UNK1:O	2.71726	Hydrogen Bond	Conventional Hydrogen Bond
	A:GLN42:HE21 - N:UNK1:O	1.90918	Hydrogen Bond	Conventional Hydrogen Bond
	A:GLN42:HE22 - N:UNK1:O	1.89367	Hydrogen Bond	Conventional Hydrogen Bond
	A:GLN42:HE22 - N:UNK1:O	2.98401	Hydrogen Bond	Conventional Hydrogen Bond
	N:UNK1:C - N:UNK1:O	3.23155	Hydrogen Bond	Carbon Hydrogen Bond
	A:HIS26:CE1 - N:UNK1:O	3.39618	Hydrogen Bond	Carbon Hydrogen Bond
	A:HIS33:CD2 - N:UNK1:O	3.41924	Hydrogen Bond	Carbon Hydrogen Bond
	A:HIS33:CD2 - N:UNK1:O	3.44672	Hydrogen Bond	Carbon Hydrogen Bond
	N:UNK1:C - A:ARG38	4.17625	Hydrophobic	Alkyl

	N:UNK1:C - A:LYS39	4.27379	Hydrophobic	Alkyl
