# Supplementary material for: Integrated In vitro and in vivo evaluation of anticancer potentials of two clerodane diterpenes isolated from Psidium guajava (L.) through molecular docking approaches
Source: PLoS One. 2026 Nov 13;21(11):e0362655. doi: 10.1371/journal.pone.0362655 (PMC13766287; doi:10.1371/journal.pone.0362655)
Supplement: Supplementary file 8 [file pone.0362655.s008.pdf]

Compound	Bond (AA and Ligand)	Bond length	Bond type	Bond nature
Borapetoside F	N:UNK1:H - A:HIS141:NE2	1.99511	Hydrogen Bond	Conventional Hydrogen Bond
	N:UNK1:H - A:LEU207:O	2.86672	Hydrogen Bond	Conventional Hydrogen Bond
	A:ARG54:HH22 - N:UNK1:O	3.01497	Hydrogen Bond	Conventional Hydrogen Bond
	A:TYR57:HH - N:UNK1:O	2.18812	Hydrogen Bond	Conventional Hydrogen Bond
	A:SER208:CB - N:UNK1:O	3.26885	Hydrogen Bond	Carbon Hydrogen Bond
	A:LYS241:CE - N:UNK1:O	3.42486	Hydrogen Bond	Carbon Hydrogen Bond
	A:LYS241:NZ - N:UNK1	3.71035	Electrostatic	Pi-Cation
	N:UNK1 - A:LYS241	4.80652	Hydrophobic	Pi-Alkyl
	A:TYR57 - N:UNK1	5.47041	Hydrophobic	Pi-Alkyl
Borapetoside B	N:UNK1:H - N:UNK1:O	2.46145	Hydrogen Bond	Conventional Hydrogen Bond
	A:LYS144:HN - N:UNK1:O	2.33551	Hydrogen Bond	Conventional Hydrogen Bond
	A:SER208:HN - N:UNK1:O	2.91752	Hydrogen Bond	Conventional Hydrogen Bond
	A:LYS241:HZ1 - N:UNK1:O	2.59204	Hydrogen Bond	Conventional Hydrogen Bond
	A:LYS272:HZ1 - N:UNK1:O	2.66055	Hydrogen Bond	Conventional Hydrogen Bond
	A:LYS272:HZ2 - N:UNK1:O	2.66809	Hydrogen Bond	Conventional Hydrogen Bond
	N:UNK1:C - A:TYR57	3.86611	Hydrophobic	Pi-Sigma
	N:UNK1:C - A:CYS59	5.05485	Hydrophobic	Alkyl
	N:UNK1 - A:LEU207	4.75689	Hydrophobic	Pi-Alkyl
Bleomycin	N:UNK1:H - A:LYS272:O	2.18202	Hydrogen Bond	Conventional Hydrogen Bond
	N:UNK1:H - A:TYR57:OH	2.84491	Hydrogen Bond	Conventional Hydrogen Bond
	N:UNK1:H - A:HIS141:NE2	2.48932	Hydrogen Bond	Conventional Hydrogen Bond
	N:UNK1:H - N:UNK1:O	2.21079	Hydrogen Bond	Conventional Hydrogen Bond
	N:UNK1:H - N:UNK1:O	1.72652	Hydrogen Bond	Conventional Hydrogen Bond
	A:ARG54:HH11 - N:UNK1:O	2.15712	Hydrogen Bond	Conventional Hydrogen Bond
	A:TYR57:HH - N:UNK1:O	3.09312	Hydrogen Bond	Conventional Hydrogen Bond
	A:CYS59:HG - N:UNK1:O	3.09862	Hydrogen Bond	Conventional Hydrogen Bond
	A:LYS144:HN - N:UNK1:O	2.40674	Hydrogen Bond	Conventional Hydrogen Bond
	A:LYS241:HZ2 - N:UNK1:O	2.0902	Hydrogen Bond	Conventional Hydrogen Bond
	A:LYS241:HZ3 - N:UNK1:O	1.92748	Hydrogen Bond	Conventional Hydrogen Bond
	A:ASN247:HD22 - N:UNK1:O	2.43476	Hydrogen Bond	Conventional Hydrogen Bond
	N:UNK1:C - N:UNK1:O	3.37499	Hydrogen Bond	Carbon Hydrogen Bond
	N:UNK1:C - A:LYS241:O	3.44496	Hydrogen Bond	Carbon Hydrogen Bond
	A:THR143:CA - N:UNK1:O	2.51246	Hydrogen Bond	Carbon Hydrogen Bond
	A:PRO243:CD - N:UNK1:O	3.52478	Hydrogen Bond	Carbon Hydrogen Bond
	N:UNK1:C - A:PRO243	4.51727	Hydrophobic	Alkyl
	N:UNK1:C - A:LYS272	2.87033	Hydrophobic	Alkyl
	N:UNK1:C - A:PRO243	3.35193	Hydrophobic	Alkyl
	N:UNK1:C - A:LYS144	3.17323	Hydrophobic	Alkyl
	N:UNK1:C - A:LEU207	3.724	Hydrophobic	Alkyl
	N:UNK1 - A:LYS241	5.1259	Hydrophobic	Pi-Alkyl

	N:UNK1 - A:LYS272	4.85348	Hydrophobic	Pi-Alkyl
